# Minithoracotomy versus sternotomy in mitral valve surgery: meta-analysis from recent matched and randomized studies

**DOI:** 10.1186/s13019-023-02229-x

**Published:** 2023-04-06

**Authors:** Adel Al Shamry, Margaux Jegaden, Salah Ashafy, Armand Eker, Olivier Jegaden

**Affiliations:** 1 Department of Cardiac Surgery and ICU, Saudi German Hospital, Dubai, UAE; 2 Department of Surgery, Kremlim Bicetre Hospital, Paris, France; 3 Department of Cardiac Surgery, Zayed Military Hospital, Abu Dhabi, UAE; 4 Department of Cardiac Surgery, Centre Cardio-Thoracic, Monaco, Monaco; 5 Department of Cardiac Surgery, Mediclinic Middle East, Mediclinic Airport Road Hospital, MBRU, PO Box 48481, Abu Dhabi, UAE

**Keywords:** Mitral valve surgery, Minimally invasive surgery, Minithoracotomy, Sternotomy, Meta-analysis

## Abstract

**Background:**

There is still ongoing debate about the benefits of mini-thoracotomy (MTH) approach in mitral valve surgery in comparison with complete sternotomy (STER). This study aims to update the current evidence with mortality as primary end point.

**Methods:**

The MEDLINE and EMBASE databases were searched through June 2022. Two randomized studies and 16 propensity score matched studies published from 2011 to 2022 were included with a total of 12,997 patients operated on from 2005 (MTH: 6467, STER: 6530). Data regarding early mortality, stroke, reoperation for bleeding, new renal failure, new onset of atrial fibrillation, need of blood transfusion, prolonged ventilation, wound infection, time-related outcomes (cross clamp time, cardiopulmonary bypass time, ventilation time, length of intensive care unit stay, length of hospital stay), midterm mortality and reoperation, and costs were extracted and submitted to a meta-analysis using weighted random effects modeling.

**Results:**

The incidence of early mortality, stroke, reoperation for bleeding and prolonged ventilation were similar, all in the absence of heterogeneity. However, the sub-group analysis showed a significant OR in favor of MTH when robotic enhancement was used. New renal failure (OR 1.67, 95% CI 1.06–2.62, *p* = 0.03), new onset of atrial fibrillation (OR 1.31, 95% CI 1.15–1.51, *p* = 0.001) and the need of blood transfusion (OR 1.77, 95% CI 1.39–2.27, *p* = 0.001) were significantly lower in MTH group. Regarding time-related outcomes, there was evidence for important heterogeneity of treatment effect among the studies. Operative times were longer in MTH: differences in means were 20.7 min for cross clamp time (95% CI 14.9–26.4, *p* = 0.001), 36.8 min for CPB time (95% CI 29.8–43.9, *p* = 0.001) and 37.7 min for total operative time (95% CI 19.6–55.8, *p* < 0.001). There was no significant difference in ventilation duration; however, the differences in means showed significantly shorter ICU stay and hospital stay after MTH compared to STER: − 0.6 days (95% CI − 1.1/− 0.21, *p* = 0.001) and − 1.88 days (95% CI − 2.72/− 1.05, *p* = 0.001) respectively, leading to a significant lower hospital cost after MTH compared to STER with difference in means − 4528 US$ (95% CI − 8725/− 326, *p* = 0.03).

The mid-term mortality was significantly higher after STER compared to MTH: OR = 1.50, 1.09–2.308 (95% CI), *p* = 0.01; the rate of mid-term reoperation was reported similar in MTH and STER: OR = 0.76, 0.50–1.15 (95% CI), *p* = 0.19.

**Conclusions:**

The present meta-analysis confirms that the MTH approach for mitral valve disease remains associated with prolonged operative times, but it is beneficial in terms of reduced postoperative complications (renal failure, atrial fibrillation, blood transfusion, wound infection), length of stay in ICU and in hospitalization, with finally a reduction in global cost. MTH approach appears associated with a significant reduction of postoperative mortality that must be confirmed by large randomized study.

**Supplementary Information:**

The online version contains supplementary material available at 10.1186/s13019-023-02229-x.

## Introduction

Minimally invasive mitral valve surgery with mini-thoracotomy approach (MTH) has been introduced 25 years ago [1]. Previous meta-analyses [2–5] failed to detect any positive impact of MTH on the occurrence of postoperative major adverse cardiac events in comparison with classic sternotomy approach (STER). Nowadays, mini-thoracotomy is established as a new standard for mitral valve surgery and the surgical community is far from the learning curve with this minimally invasive technique; the proponents arguing its utility for treating even the most complex mitral valve disease without any additional risk of potential complications despite prolonged operation times.

The aim of this meta-analysis based only on recent comparative series published from 2010 and including patients operated after 2005, was to investigate the early and late performance of MTH versus STER in mitral valve surgery and to detect any more substantial benefit and less drawbacks that could be expected with larger experience, larger expertise and more standardized techniques in minimally invasive approach, over time. Mortality as primary end point and major complications were the main interest; in addition procedure-related and resource-related outcomes were assessed.

## Methods

The meta-analysis was performed in accordance with PRISMA and MOOSE guidelines [6, 7]. Databases were searched for articles meeting our inclusion criteria and published by June 2022: PubMed/MEDLINE, Cochrane Controlled Trials Register (CENTRAL/CCTR), EMBASE, Google Scholar, Clinical Trials.gov. Search terms were “minimally invasive mitral”, “mitral minithoracotomy”, “less invasive mitral”, “robotic mitral”, “endoscopic mitral”, “totally endoscopic mitral”, robotically assisted mitral”, “mitral sternotomy”, and variants and combinations of these keywords.

### Inclusion criteria

Randomized controlled trials or propensity-score matched nonrandomized observational studies, comparing mitral valve surgery (repair or replacement) via a right lateral minithoracotomy (with or without robotic support) versus sternotomy (through a complete median sternotomy) were included.

### Exclusion criteria

Studies published before 2010 and studies including surgery performed before 2005 were excluded. Studies including mainly redo surgical procedures were excluded.

### End-points

End points were defined as early mortality, stroke, reoperation for bleeding, new renal failure, new onset of atrial fibrillation, need of blood transfusion, prolonged ventilation, wound infection, time-related outcomes: cross clamp time, cardiopulmonary bypass time (CPB), ventilation time, length of intensive care unit (ICU) stay, length of hospital stay, midterm mortality and reoperation, and costs.

## Study quality appraisal

Study quality of the included studies was assessed using the Risk of Bias in Non-randomized Studies of Interventions (ROBINS-I) tool [8]. Using this tools, seven domains of bias were assessed and each study was then classified as either low, moderate, serious or critical risk. Quality appraisal was undertaken independently by two reviewers (AA & OJ).

### Data analysis

Baseline characteristics were checked by two independent reviewers in each selected study to assess the balance in randomization or matching and the associated risk of bias. For studies reporting interquartile ranges, the mean and standard deviation were estimated according to appropriate formula [9]. Funnel plot was used to evaluate publication bias statistically analyzed by Egger’s test. The χ^2^ test and I^2^ test were used to assess study heterogeneity; if heterogeneity was significant (I^2^ > 75%), the analysis used a random effects model. Odds ratio (OR) with 95% confidence interval (CI) were calculated for discrete data. For continuous data, differences in means with 95% CI were considered. *p*-values < 0.05 were considered statistically significant. The OR and differences in means were combined across the studies using a weighted random effects model. A sub-group analysis regarding robotic-enhancement was added. Forest plots of log-OR were used to represent the synthesis of the results when appropriate. The analysis and data modelling were performed with the IBM-SPSS statistics software version 28.0 (IBM-SPSS Inv, Armonk, NY).

## Results

### Study selection

**Fig. 1:**
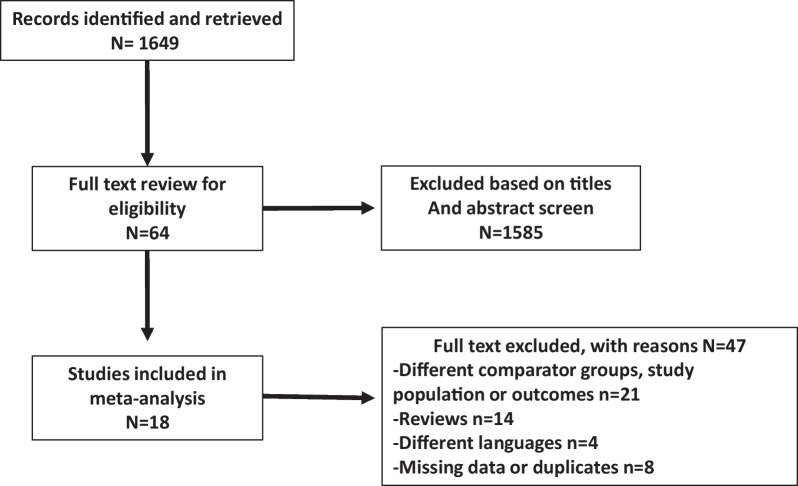
Flow chart of the study

A total of 1649 citations were identified, of which 64 studies were potentially relevant and retrieved for full review. Eighteen articles included studies that met our eligibility criteria for the comparison of MTH versus STER (Fig. 1). Two studies [11, 24] were prospectively randomized; the other 16 studies [10, 12–23, 25–27] were nonrandomized, retrospective and propensity score matched. The quality of the included nonrandomized studies, which was assessed using the ROBINS-I tool, was deemed to be low risk of bias in six studies, moderate in six studies and serious in four (Additional file 1). Four studies were multicentric, nine unicentric and five from database (Table 1). A robotic-enhancement for MTH was used in 6 studies [22–27].

**Table 1:** Study characteristics of relevant articles identified for meta-analysis

Author	Date of publication	Study period	Origin of series	Propensity matched (PM) or Randomized (Rand)	Sternotomy approach (N)	Mini-thoracotomy approach (N)	Robotic Enhancement (Y/N)
Grossi et al. [10]	2014	2007–2011	Database	PM	367	367	N
Nasso et al. [11]	2014	2008–2013	Multicentric	Rand	80	80	N
Nishi et al. [12]	2015	2008–2012	Database	PM	750	750	N
Downs et al. [13]	2016	2011–2014	Database	PM	355	355	N
Hawkins et al. [14]	2018	2011–2016	Unicentric	PM	74	74	N
Wang Q et al. [15]	2018	2012–2015	Unicentric	PM	67	67	N
Grant et al. [16]	2019	2008–2016	Multicentric	PM	639	639	N
Liu et al. [17]	2019	2012–2015	Unicentric	PM	202	202	N
Paparella et al. [18]	2020	2011–2017	Multicentric	PM	1493	1493	N
Cetinkaya et al. [19]	2021	2005–2015	Unicentric	PM	422	422	N
Pojar et al. [20]	2021	2012–2018	Unicentric	PM	225	158	N
Olsthoorn et al. [21]	2022	2013–2018	Multicentric	PM	718	718	N
Mihaljevic et al. [22]	2011	2006–2009	Unicentric	PM	106	106	Y
Suri et al. [23]	2011	2007–2010	Unicentric	PM	95	95	Y
Iyigun et al. [24]	2017	2013–2015	Unicentric	Rand	29	33	Y
Hawkins et al. [25]	2018	2011–2016	Database	PM	314	314	Y
Wang A et al. [26]	2018	2011–2014	Database	PM	503	503	Y
Coyan et al. [27]	2018	2013–2015	Unicentric	PM	91	91	Y

### Baseline characteristics

A total of 12,997 patients operated on from 2005 (MTH: 6467, STER: 6530) were included from studies published from 2011 to 2022. The baseline characteristics of patients are summarized in Table 2, by study, regardless the surgical technique performed, to identify the population treated. Globally the populations were homogenous: a young population (mean age 59 years), mainly in functional class 1 or 2, with a preserved left ventricular (LV) function (mean LV ejection fraction from 56 to 65%), and a low incidence of cerebro-vascular event or coronary artery disease. Wang A et al. [26] reported an older population with mean age 71 years, but without other significant risk factors. These characteristics defined a population with a low risk for mitral valve surgery as it was confirmed by risk scores when they were available (Table 3). The mitral surgery performed was mainly mitral repair. In only two studies [17, 24], the rate of valve replacement was > 30%, but well balanced in randomization or propensity score matching. An associated tricuspid valve repair was frequent in 3 studies [15, 17, 20] and an associated atrial fibrillation surgery was reported higher than 20% in 5 studies (Table 3); both associated procedures represented a moderate risk of bias because they were well balanced in propensity score matching.

**Table 2:** Summary of baseline characteristics in patient populations

Author	Age	Male Gender (%)	NYHA class 3–4 (%)	Hypertension (%)	Coronary Disease (%)	Cerebro-vascular events (%)	Atrial fibrillation (%)	LV impairment (%)	LVEF (%)
Grossi et al. [10]	65*	56.1	NI	NI	NI	NI	NI	NI	NI
Nasso et al. [11]	54.1 ± 10.5	56.8	26.8	NI	NI	NI	NI	26.8	NI
Nishi et al. [12]	55.5 ± 12.6	59.8	11.3	41.6	NI	2.8	NI	11	NI
Downs et al. [13]	58.1 ± 13.5	60.6	19.4	56.8	NI	NI	NI	NI	57.9 ± 9.6
Hawkins et al. [14]	61.6 ± 13.8	57.4	NI	60.1	14.1	10.1	39.9	NI	60
Wang Q et al. [15]	51 ± 12	51.5	26.9	22.4	NI	2.2	39.5	NI	55.9 ± 12
Grant et al. [16]	62.8 ± 12.6	66.4	47.6	45.6	5.9	2.7	33.6	17.5	NI
Liu et al. [17]	50.7 ± 11.5	34.6	26.9	6.9	2.2	6.7	49.2	NI	62.8 ± 7.9
Paparella et al. [18]	66.5 ± 12	48.9	NI	62	6.5	1	29.2	26.6	NI
Cetinkaya et al. [19]	64.1 ± 12.7	54.7	84.8	49.5	7.7	5.2	33.4	NI	57.5*
Pojar et al. [20]	65.1 ± 10.1	41.5	43.3	73	NI	8.1	44.3	NI	58.4 ± 10.7
Olsthoorn et al. [21]	63.6 ± 12	57	NI	NI	NI	NI	NI	14.6	NI
Mihaljevic et al. [22]	61 ± 11	75	20	46	NI	1.8	7	NI	NI
Suri et al. [23]	55.3 ± 12.6	78.4	10	32.1	1.6	0.5	4.7	NI	65.3 ± 6.2
Iyigun et al. [24]	49.9 ± 13.7	32.2	NI	NI	NI	NI	NI	NI	NI
Hawkins et al. [25]	61*	58.1	NI	63.2	14.9	6.2	10.8	NI	60*
Wang A et al. [26]	71 ± 5	61	44.9	68.2	7.4	3.4	14.4	NI	59 ± 8
Coyan et al. [27]	62*	56	32	57	NI	10	26	NI	59*

NYHA, New York heart association; LV, left ventricle; LVEF, left ventricular ejection fraction; NI, not indicated

*Median

**Table 3:** Summary of risk scores, surgical techniques performed and follow-up in patient populations

Author	Risk score	MV Repair (%)	MV replacement (%)	Associated TV repair (%)	Associated AF surgery (%)	Follow-up (years)
Grossi et al. [10]	NI	100	0	NI	NI	NI
Nasso et al. [11]	NI	100	0	NI	NI	3.2 ± 1.4
Nishi et al. [12]	NI	100	0	NI	NI	NI
Downs et al. [13]	1.75 ± 3.9^a^	77.9	22.1	NI	27.3	NI
Hawkins et al. [14]	NI	73.3	26.7	9.4	37.8	NI
Wang Q et al. [15]	3.17 ± 1.2^b^	100	0	70.1	23.9	2.8*
Grant et al. [16]	5.4 ± 5.7^c^	84.1	15.9	8.05	17.1	3.7*
Liu et al. [17]	1.34 ± 0.67^d^	0	100	93.8	NI	2.2 ± 1.1
Paparella et al. [18]	2.6 ± 2.96^d^	65.2	34.8	13.7	NI	NI
Cetinkaya et al. [19]	7.6 ± 10.3^c^	86.75	13.25	16.65	26.4	3
Pojar et al. [20]	2.78 ± 2.4^d^	83.8	16.2	49.3	46.7	3.6 ± 2.1
Olsthoorn et al. [21]	3^b^*	78.6	21.4	10.7	14.1	3.2 ± 2
Mihaljevic et al. [22]	NI	100	0	NI	NI	NI
Suri et al. [23]	NI	100	0	NI	NI	NI
Iyigun et al. [24]	NI	32.2	64.8	12.9	NI	NI
Hawkins et al. [25]	0.6^a^*	82.9	17.1	NI	NI	NI
Wang A et al. [26]	2 ± 2^a^	94.4	5.6	NI	NI	1.8 ± 1.2
Coyan et al. [27]	0.8^a^*	83.5	16.5	NI	NI	NI

*MV* Mitral valve, *TV* Tricuspid valve, *AF* Atrial fibrillation, *NI* Not indicated

*Median

^a^STS PROM score

^b^Euroscore

^c^logistic Euroscore

^d^Euroscore 2

### Mortality

**Fig. 2:**
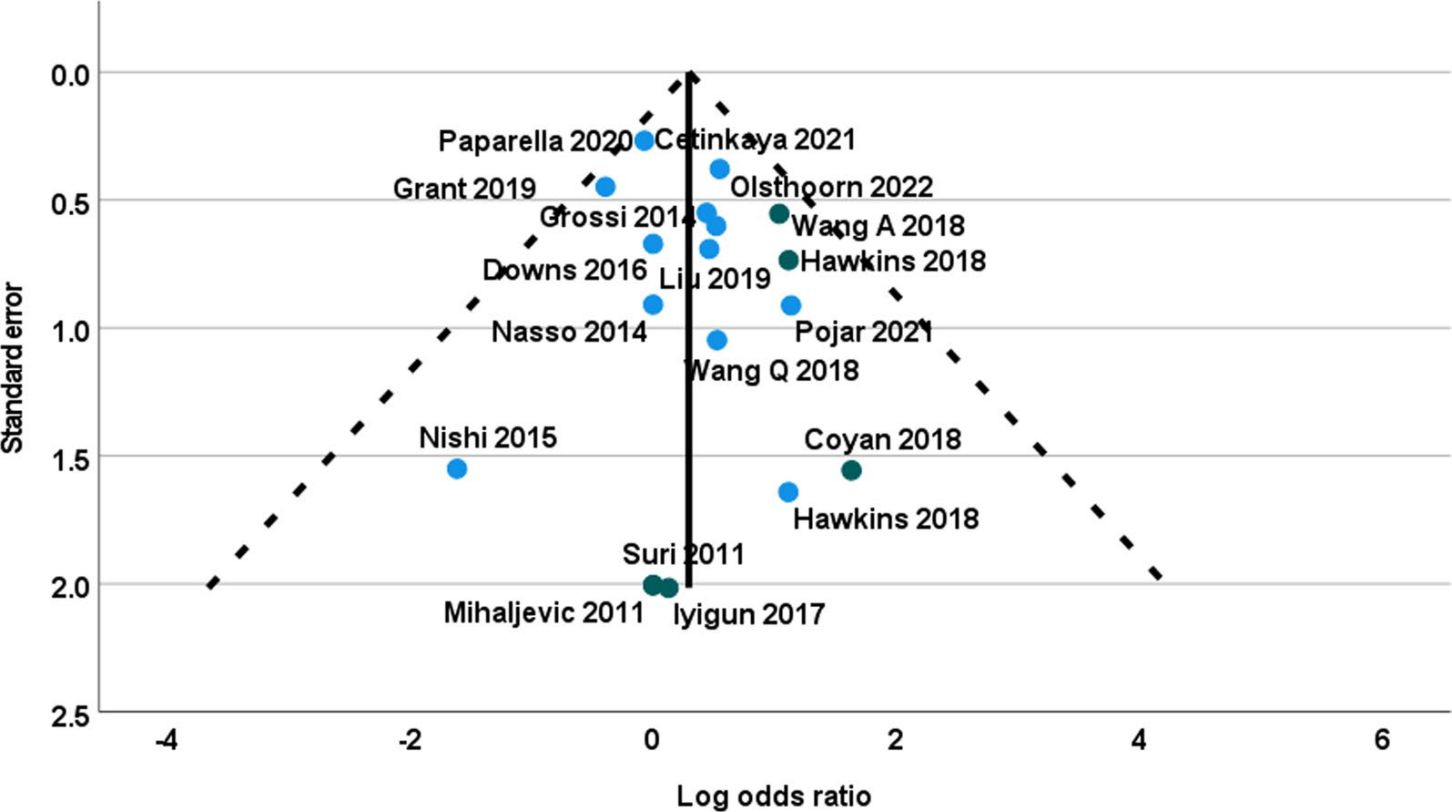
Funnel plot of early mortality (Log odds ratio)

**Fig. 3:**
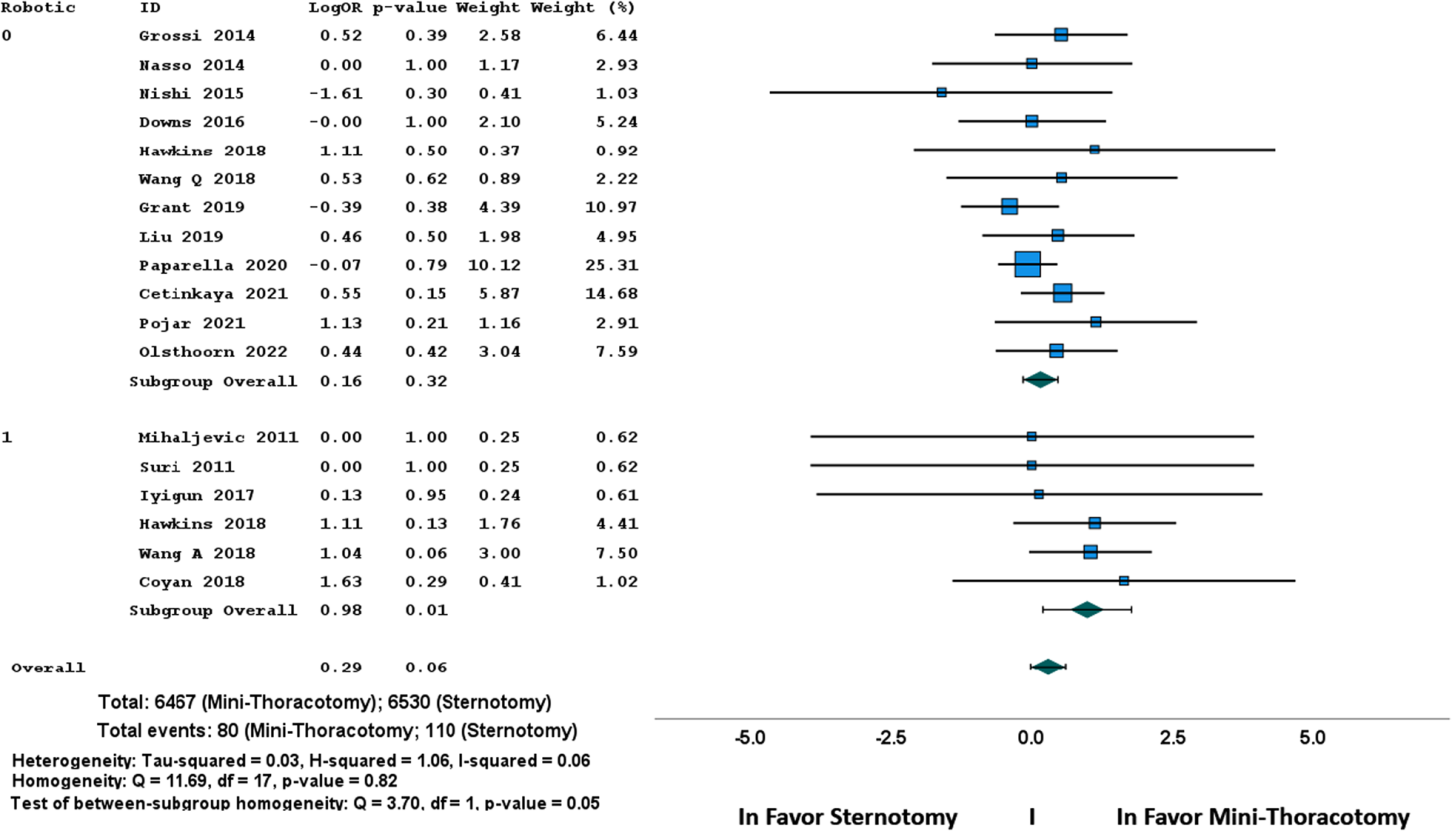
Forest plot of early mortality (Odds ratio)

Early mortality was described in all the 18 studies; it was 1.48% and it was significantly lower in patients treated with MTH than in patients treated with STER (1.23% vs 1.63% respectively, χ^2^ = 4.51, *p* = 0.033). There was no heterogeneity among the studies (Fig. 2) and the funnel plot showed no asymmetry (Fig. 3). However, the overall OR of early mortality showed no difference between MTH and STER: OR = 1.37, 0.96–1.92 (95% CI), *p* = 0.06 (Table 4). The sub-group analysis showed a significant OR in favor of MTH when robotic enhancement was used (Fig. 2, Table 4).

**Table 4:** Summary of postoperative complications

Complications	N	Sample size/Events		Statistics		Heterogeneity		Sub-group homogeneity	Publication bias
		MiniTh (n)	Sternotomy (n)	OR	95% CI *p*-value	χ^2^ test *p*-value	I^2^ test (%)	Q test *p*-value	Eggers test *p*-value
Mortality	18	6467 80	6530 110	1.37	0.96–1.92 0.06	0.76	8.6	0.05	0.850
Stroke	16	6367 68	6434 85	1.23	0.89–1.70 0.21	0.99	0	0.69	0.159
Reoperation for bleeding	14	5565 192	5632 180	0.91	0.73–1.12 0.38	0.41	0	0.43	0.765
Renal failure	14	5881 132	5948 202	1.67	1.06–2.62 0.03	0.04	46	0.81	0.276
Atrial fibrillation	14	5348 973	5415 1232	1.31	1.15–1.51 0.001	0.20	37	0.74	0.108
Blood transfusion	12	4756 1155	4823 1557	1.77	1.39–2.27 0.001	0.001	81	0.86	0.642
Prolonged ventilation	10	3140 96	3207 120	1.22	0.80–1.88 0.36	0.07	47	0.31	0.987
Wound infection	10	4415 38	4482 75	1.86	1.17–2.96 0.01	0.72	11	0.73	0.978
Mid-term mortality	8	2519 107	2555 164	1.50	1.09–2.08 0.01	0.51	24	0.72	0.174
Mid-term reoperation	7	2059 56	2106 45	0.76	0.50–1.15 0.19	0.65	0	0.41	0.266

*N* Number of series, *MiniTh* Mini-thoracotomy, *CI* Confidence interval, *OR* Odds ratio

### Stroke

Total stroke rate was 1.2% in 16 studies (NA: 15, 24) and 12,801 patients, without difference between groups. The overall OR of stroke was 1.23 without difference between MTH and STER (95% CI 0.89–1.70, *p* = 0.21). There was no heterogeneity and sub-group analysis according to the robotic enhancement did not differ (Table 4).

### Other complications

All other complications are summarized in Table 4. Reoperation for bleeding and prolonged ventilation were observed without difference between groups, and with a non-significant overall OR. New renal failure, new onset of atrial fibrillation and the need of blood transfusion were significantly lower in MTH group than in STER group with significant ORs. In 14 studies (NA: 10, 11, 22, 24) and 11,829 patients, overall OR of new renal failure was 1.67 (95% CI 1.06–2.62, *p* = 0.03). In 14 studies (NA: 10, 11, 16, 24) and 10,763 patients, overall OR of new onset of atrial fibrillation was 1.31 (95% CI 1.15–1.51, *p* = 0.001). In 12 studies (NA: 10, 11, 19, 24, 27) and 9579 patients, overall OR of blood transfusion requirement was 1.77 (95% CI 1.39–2.21, *p* = 0.001). The occurrence of wound infection was lower in MTH approach and overall OR was 1.86 (95% CI 1.17–2.96, *p* = 0.01). There was significant heterogeneity among the studies in new renal failure and blood transfusion with moderate disparity, as observed in funnel and forest plots (Additional file 2). The sub-group analysis according to the robotic enhancement did not differ in results (Table 4).

### Time related outcomes

Time related data are reported in Table 5. Cross clamp time and CPB time were significantly longer after MTH approach compared to STER approach; 16 studies (NA: 8) reported the data of 10,827 patients. The overall differences in means according to random-effects model were 20.7 min for cross clamp time (95% CI 14.9–26.4, *p* = 0.001) and 36.8 min for CPB time (95% CI 29.8–43.9, *p* = 0.001); a significant publication bias was detected for both criteria, it was related to the overstatement of the difference in one series with robotic enhancement (Additional file 2) and it did not justify the withdrawal of the series [15].

**Table 5:** Summary of time-related outcomes and costs

Outcomes	N	Sample size		Statistics		Heterogeneity		Sub-group homogeneity	Publication bias
		MiniTh (n)	Sternotomy (n)	Diff. in means	95% CI *p*-value	χ^2^ test *p*-value	I^2^ test (%)	Q test *p*-value	Eggers test *p*-value
Cross-Clamp time, min	16	5382	5445	20.7	14.9/26.4 0.001	0.001	96	0.87	0.001
CPB time, min	16	5382	5445	36.8	29.8/43.9 0.001	0.001	95	0.67	0.001
Total operative time, min	9	2173	2240	37.7	19.6/55.8 0.001	0.001	97	0.001	0.193
Ventilation time, hrs	8	1453	1520	− 4.6	− 10.6/1.4 0.13	0.001	99	0.001	0.469
ICU stay, days	14	4637	4700	− 0.6	− 1.1/− 0.21 0.001	0.001	98	0.48	0.473
Hospital stay, days	17	6100	6163	− 1.88	− 2.72/− 1.05 0.001	0.001	97	0.97	0.458
Total cost (USD)	5	1045	1112	− 4525	− 8725/− 326 0.03	0.001	92	0.13	0.222

*N* Number of series, *MiniTh* Mini-thoracotomy, *Diff* Difference, *CI* Confidence interval, *CPB* Cardio-pulmonary bypass, *ICU* Intensive care unit, *USD* US dollar

The overall differences in means showed a significantly longer total operative time in MTH compared to STER: 37.7 min (95% CI 19.6–55.8, *p* = 0.001), and robotic enhancement made the difference greater implying a significant sub-group heterogeneity (Table 4, Additional file 2). There was no significant difference in ventilation duration. However, the overall differences in means showed a significantly shorter ICU stay after MTH compared to STER: − 0.6 days (95% CI − 1.1/− 0.21, *p* = 0.001), in 14 studies (NA: 10, 16, 22) and 9337 patients, and a significantly shorter hospital stay after MTH compared to STER: − 1.88 days (95% CI − 2.72/− 1.05, *p* = 0.001), in 17 studies (NA: 10) and 12,263 patients. There were evidences for important heterogeneity of treatment effect in time-related criteria among the studies (Table 4), but the disparities were always in the same side as observed in funnel and forest plots (Additional file 2) and there were well compensated by the random-effects model used.

### Inhospital cost

Total cost of both procedures was reported in 5 studies [10, 13, 14, 20, 27] and 2157 patients (Table 5). The overall difference in means showed a significant lower cost after MTH compared to STER: − 4528 US$ (95% CI − 8725/− 326, *p* = 0.03). The sub-group analysis according to the robotic enhancement did not differ in results (Table 5).

### Long-term outcomes

Mid-term mortality was reported in 8 studies [11, 15–17, 19–21, 26] and 5074 patients within 3-year mean follow-up, from 1.8 to 3.6 years (Table 3). There was no evidence of heterogeneity of treatment effect among the studies (Additional file 2). The overall OR of mid-term mortality showed a significant higher rate after STER compared to MTH: OR = 1.50, 1.09–2.08 (95% CI), *p* = 0.01 (Table 4). The sub-group analysis according to the robotic enhancement did not differ in results.

Rate of mid-term reoperation was reported in 7 studies [11, 15–17, 20, 21, 26] and 4165 patients within a 2.8-year mean follow-up, from 1.8 to 3.6 years (Table 3). There was no difference between MTH and STER groups (Table 4): overall OR was 0.76 (95% CI 0.50–1.15, *p* = 0.19).

## Discussion

The benefits of minimally invasive approach via mini-thoracotomy in mitral valve surgery remains controversial, when compared to conventional approach via a sternotomy. It is currently unclear whether the potential benefits of MTH outweigh its disadvantages or drawbacks. Previous meta-analyses were mainly based on historical series, including learning curve, regardless the evolution of the surgical technique itself and the improvement of the tools dedicated to a minimally invasive environment; the main differences between the two approaches were found for procedure and resource related outcomes [2–5]. These outcomes are often used to argue for or against one or the other procedure. Nowadays, mini-thoracotomy approach is established as a new standard for mitral valve surgery with dedicated tools and techniques [28]. The aim of this meta-analysis based only on recent randomized or matched series published from 2010 and including patients operated after 2005, was to identify substantial benefits on perioperative outcomes when standardized MTH approach and mitral valve operation were performed.

In line with previous reports [4, 5], we observed no difference regarding early mortality and major postoperative complications as stroke, reoperation for bleeding, or prolonged ventilation. However, a trend towards a lower early mortality in MTH approach was observed with a significant difference in basic tests (*p* = 0.03) that was not confirmed in the weighted random effects model analysis (*p* = 0.06). Interestingly, the sub-group analysis showed a significant OR in favor of MTH when robotic enhancement was used (*p* = 0.01); this result is mainly related to two database series [25, 26] and must be carefully interpreted (Figs. 2 and 3); it has been reported by Williams ML et al. [29] in a previous meta-analysis based on the same series and it needs further confirmation. The benefits of MTH appear limited in a significant lower rate of renal failure, of new onset of atrial fibrillation and a significant lower requirement of blood transfusion. Despite having longer operative times (clamp time, CPB time and total operations time), MTH was associated with significant shorter lengths of ICU and hospital stay, and finally a significant reduction in the mean hospitalization cost. These results in time-related outcomes are consistent and possibly correlated together: lower incidence of renal failure, of atrial fibrillation and blood transfusion may contribute to lower lengths of stay and costs.

Interestingly, some drawbacks of the minimally invasive approach previously reported [2, 3] have been less observed and reported in this meta-analysis; there is no more an additional risk of stroke or vascular complication in MTH approach compared with conventional STER, probably thanks to the standardization of the technique that made this approach safer [30, 31].

According to this meta-analysis, the benefit impact of MTH in comparison of sternotomy remains limited. Consequently, in one hand the advantages of MTH are not strong enough to convince surgeons to change their practice and in the other hand the benefits were observed in a low risk population and are too limited to contribute to extending the indications to a higher risk population for whom longer operative times represent an obvious potential risk of increased complications [32, 33]. That remains the dilemma of minimally invasive approach in mitral valve surgery, more than 20 years after its introduction. The robotic-enhancement of the technique that is becoming a new standard [34] does not contribute to solve the problem; the sub-group analysis done in our study was not able to identify a difference in results, except for early mortality with the limitations mentioned previously, in line with previous report [29, 35].

However, indications for mitral valve surgery are recommended at the early stage of the disease, especially in mitral regurgitation [36, 37], representing a low risk population, more often asymptomatic or pauci-symptomatic patients who are demanding mini surgical access; that contributes to the diffusion of MTH approach. Moreover, if the tendency to a lower mortality is confirmed in the next future it could contribute to a better adoption rate of the MTH approach as well.

The meta-analysis reported by Moscarelli et al. [38] showed that the inclusion of high risk patients has not compromised the expected results of mini-access and it could be time to explore the advantages of MTH approach in high risk populations for mitral valve surgery. Possibly, the multicentric randomized controlled trial in process in UK (UK mini mitral) could modify the debate in a next future [39].

In this meta-analysis the midterm mortality was reported significantly lower in MTH approach; this result is based on eight studies with risk of bias due to confounding moderate in two and severe in three and it must be considered carefully. However, we can speculate that a lower rate of renal failure, atrial fibrillation, wound infection and even blood transfusion could have an impact on the midterm mortality that was reported within 3 year mean follow-up in studies. The midterm rate of reoperation was reported low and similar in both groups, confirming the durability of the results with both approaches [40].

## Limitations

The present study had several limitations. Different pathologies and techniques were reported and may have increased the level of clinical heterogeneity among studies; however, it was part of the selection criteria to verify if this heterogeneity had been well balanced in randomization or propensity score matching. Nevertheless, it was not possible to analyze the heterogeneity of the techniques of repair among the series. It was a choice in the design of the study to consider MTH as the concept of mini-approach and robotic- or video-assistance as tools; however a sub-group analysis was included to detect any specific impact of robotic enhancement. Femoral cannulation was a criteria of inclusion, however, the techniques of cross clamp were variable (transthoracic or endoaortic). Nowadays, both technique are equivalent after some learning curve [30, 31]. Regarding the analysis of the outcomes, the completeness of the series was not enough to pool the events in a “MACE index”, in reference with STS complications, and they were reported separately. The statistical heterogeneity in outcome was moderate, mainly reported in time-related outcomes and it was counterbalanced by using weighted random effects model. Finally forest and funnel plots for significant OR were reported in Additional file 2 to illustrate the possible bias across studies and results. Definition of early mortality and follow-up for midterm results were changing according to studies and it led to being careful in the interpretation of the results regarding early and midterm mortality, regardless risk of bias due to confounding that has been reported above.

## Conclusion

The present meta-analysis confirmed that the MTH approach for mitral valve disease has remained associated with prolonged operative times but it was beneficial in terms of reduced postoperative complications (renal failure, atrial fibrillation, blood transfusion, wound infection), length of stay in ICU and in hospitalization, with finally a reduction in global cost. This limited impact may explain that in daily practice, MTH approach remain performed mainly in low risk patient to avoid any additional risk related to longer operative times in patient with a more severe profile related to cardiac or non-cardiac risk factors. However, this meta-analysis detected that MTH approach could be associated with a significant reduction of postoperative early and midterm mortality that must be confirmed by large randomized study but it may open the way to a new era demonstrating that benefits of MTH outweigh its drawbacks. Finally, in this meta-analysis there was no evidence of any additional benefit from robotic enhancement in MTH approach for mitral valve surgery; but that needs to be analyzed in a dedicated randomized study.

## Supplementary Information

**Additional file 1**. Risk of bias in non-randomized studies according to ROBINS-I tool.

**Additional file 2**. Forest plots of the criteria analyzed with respective funnel plots if significant Odds ratio.

## Data Availability

The data sets used and analysed during the current study are available from the corresponding author.
