# Supplementary material for: Minithoracotomy versus sternotomy in mitral valve surgery: meta-analysis from recent matched and randomized studies
Source: J Cardiothorac Surg. 2023 Apr 6;18:101. doi: 10.1186/s13019-023-02229-x (PMC10080824; doi:10.1186/s13019-023-02229-x)
Supplement: Supplementary file 1 — Additional file 1. Risk of bias in non-randomized studies according to ROBINS-I tool. [file 13019_2023_2229_MOESM1_ESM.docx]

**Additional table**: Risk of bias in non-randomized studies according to ROBINS-I tool.

| Study ID | A | B | C | D | E | F | G |
| --- | --- | --- | --- | --- | --- | --- | --- |
| Grossi et al (8) | **2** | **1** | **1** | **1** | **2** | **1** | **1** |
| Nishi et al (10) | **1** | **1** | **1** | **1** | **1** | **1** | **1** |
| Downs et al (11) | **2** | **1** | **1** | **1** | **1** | **1** | **1** |
| Hawkins et al (12) | **2** | **1** | **1** | **1** | **1** | **1** | **1** |
| Wang Q et al (13) | **3** | **1** | **1** | **1** | **1** | **1** | **1** |
| Grant et al (14) | **2** | **1** | **1** | **1** | **1** | **1** | **1** |
| Liu et al (15) | **3** | **1** | **1** | **1** | **1** | **1** | **1** |
| Paparella et al (16) | **2** | **1** | **1** | **1** | **1** | **1** | **1** |
| Cetinkaya et al (17) | **3** | **1** | **1** | **1** | **1** | **1** | **1** |
| Pojar et al (18) | **3** | **1** | **1** | **1** | **1** | **1** | **1** |
| Olsthoorn et al (19) | **2** | **1** | **1** | **1** | **1** | **1** | **1** |
| Mihaljevic et al (20) | **1** | **1** | **1** | **1** | **1** | **1** | **1** |
| Suri et al (21) | **1** | **1** | **1** | **1** | **1** | **1** | **1** |
| Hawkins et al (23) | **1** | **1** | **1** | **1** | **1** | **1** | **1** |
| Wang A et al (24) | **1** | **1** | **1** | **1** | **1** | **1** | **1** |
| Coyan et al (25) | **1** | **1** | **1** | **1** | **1** | **1** | **1** |

A, Bias due to confounding; B, Bias in selection of participants into the study; C, Bias in classification of interventions; D, Bias due to deviations from intended interventions; E, Bias due to missing data; F, Bias in measurement of outcomes; G, Bias in selection of the reported result.

0, no information; 1, low risk; 2, moderate risk; 3, serious risk; 4, critical risk.
