# Supplementary material for: Minithoracotomy versus sternotomy in mitral valve surgery: meta-analysis from recent matched and randomized studies
Source: J Cardiothorac Surg. 2023 Apr 6;18:101. doi: 10.1186/s13019-023-02229-x (PMC10080824; doi:10.1186/s13019-023-02229-x)

Forest Plot - Stroke

Robotic	ID	LogOR	Lower	Upper	p-value	Weight	Weight (%)
0	Grossi 2014	-0.12	-1.08	0.84	0.81	4.14	11.29
	Nasso 2014	1.11	-2.10	4.33	0.50	0.37	1.01
	Nishi 2015	0.00	-1.14	1.14	1.00	2.98	8.12
	Downs 2016	0.00	-1.61	1.61	1.00	1.49	4.06
	Hawkins 2018	-1.11	-4.33	2.10	0.50	0.37	1.01
	Grant 2019	0.29	-0.58	1.16	0.51	5.06	13.80
	Liu 2019	0.41	-1.39	2.21	0.65	1.19	3.23
	Paparella 2020	0.13	-0.88	1.15	0.80	3.71	10.13
	Cetinkaya 2021	0.59	-0.24	1.42	0.16	5.60	15.28
	Pojar 2021	-0.07	-1.58	1.44	0.93	1.68	4.59
	Olsthoorn 2022	0.70	-0.51	1.90	0.26	2.65	7.22
	Subgroup Overall	0.24	-0.12	0.60	0.19		
1	Mihaljevic 2011	-0.70	-3.12	1.71	0.57	0.66	1.80
	Suri 2011	-1.11	-4.32	2.10	0.50	0.37	1.01
	Hawkins 2018	-0.29	-1.80	1.21	0.70	1.70	4.62
	Wang A 2018	0.46	-0.50	1.42	0.35	4.20	11.47
	Coyan 2018	0.00	-2.79	2.79	1.00	0.49	1.35
	Subgroup Overall	0.08	-0.64	0.80	0.84		
Overall		0.21	-0.12	0.53	0.21		

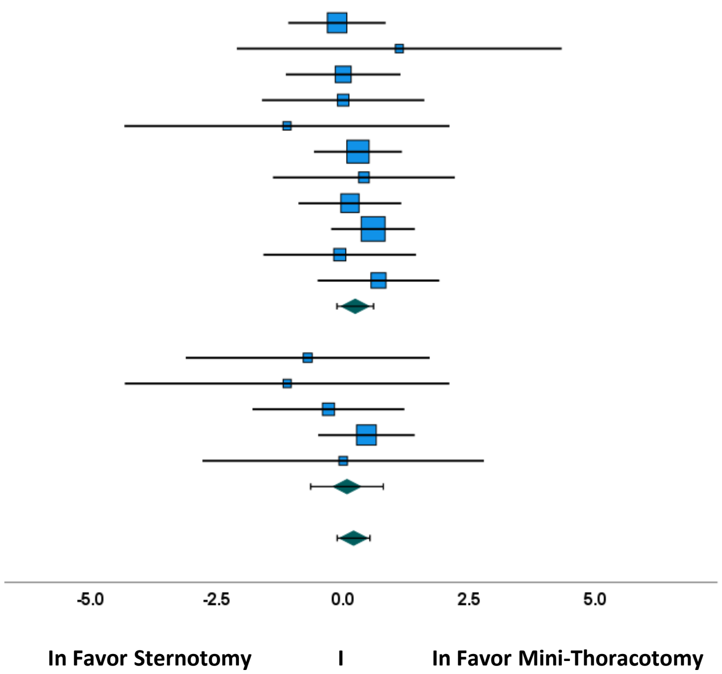

Model: Random-effects model
Heterogeneity: Tau-squared = 0.00, H-squared = 1.00, I-squared = 0.00
Homogeneity: Q = 5.19, df = 15, p-value = 0.99
Test of between-subgroup homogeneity: Q = 0.16, df = 1, p-value = 0.69

Forest Plot - Reoperation for bleeding

Robotic	ID	LogOR	Lower	Upper	p-value	Weight	Weight (%)
0	Nishi 2015	-0.91	-1.69	-0.13	0.02	6.28	7.38
	Downs 2016	-0.23	-1.01	0.54	0.56	6.42	7.55
	Hawkins 2018	-0.71	-3.13	1.72	0.57	0.65	0.77
	Wang Q 2018	-0.72	-2.46	1.01	0.41	1.28	1.51
	Grant 2019	0.10	-0.41	0.62	0.69	14.49	17.04
	Liu 2019	-0.42	-1.70	0.86	0.52	2.34	2.76
	Paparella 2020	-0.00	-0.45	0.45	1.00	18.99	22.34
	Pojar 2021	1.09	0.09	2.10	0.03	3.83	4.50
	Olsthoorn 2022	-0.03	-0.49	0.43	0.91	18.21	21.42
	Subgroup Overall	-0.06	-0.29	0.17	0.61		
1	Mihaljevic 2011	0.00	-1.41	1.41	1.00	1.92	2.26
	Suri 2011	0.70	-1.71	3.12	0.57	0.66	0.77
	Hawkins 2018	-0.29	-1.36	0.78	0.59	3.35	3.94
	Wang A 2018	-0.42	-1.23	0.39	0.31	5.86	6.89
	Coyan 2018	-1.12	-3.40	1.16	0.34	0.74	0.87
	Subgroup Overall	-0.30	-0.86	0.25	0.28		
Overall		-0.10	-0.31	0.12	0.38		

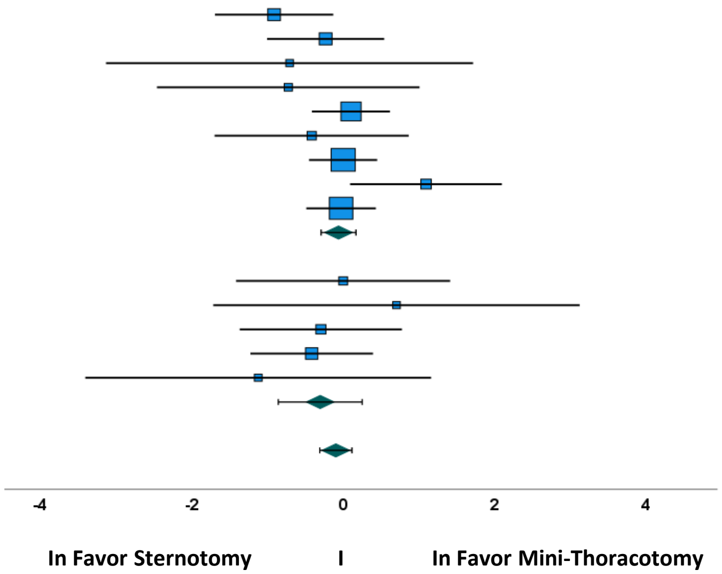

Model: Random-effects model
Heterogeneity: Tau-squared = 0.00, H-squared = 1.00, I-squared = 0.00
Homogeneity: Q = 13.48, df = 13, p-value = 0.41
Test of between-subgroup homogeneity: Q = 0.62, df = 1, p-value = 0.43

Robotic	ID	LogOR	Lower	Upper	p-value	Weight	Weight (%)
0	Nishi 2015	0.32	-0.59	1.24	0.49	2.11	11.21
	Downs 2016	-0.70	-2.40	1.00	0.42	0.99	5.26
	Hawkins 2018	0.42	-1.40	2.24	0.65	0.89	4.76
	Wang Q 2018	0.00	-1.99	1.99	1.00	0.78	4.14
	Grant 2019	0.29	-0.77	1.36	0.59	1.81	9.66
	Liu 2019	1.96	-1.01	4.93	0.20	0.39	2.09
	Paparella 2020	0.11	-0.18	0.41	0.45	3.58	19.07
	Cetinkaya 2021	1.82	1.10	2.54	0.00	2.55	13.57
	Pojar 2021	0.05	-1.23	1.34	0.93	1.46	7.79
	Olsthoorn 2022	0.85	-0.50	2.21	0.22	1.36	7.24
	Subgroup Overall	0.50	-0.02	1.02	0.06		
1	Suri 2011	1.11	-2.10	4.32	0.50	0.34	1.81
	Hawkins 2018	-0.70	-3.10	1.71	0.57	0.57	3.02
	Wang A 2018	0.82	-0.36	2.01	0.17	1.61	8.57
	Coyan 2018	1.10	-2.12	4.31	0.50	0.34	1.81
	Subgroup Overall	0.63	-0.33	1.59	0.20		
Overall		0.51	0.06	0.96	0.03		

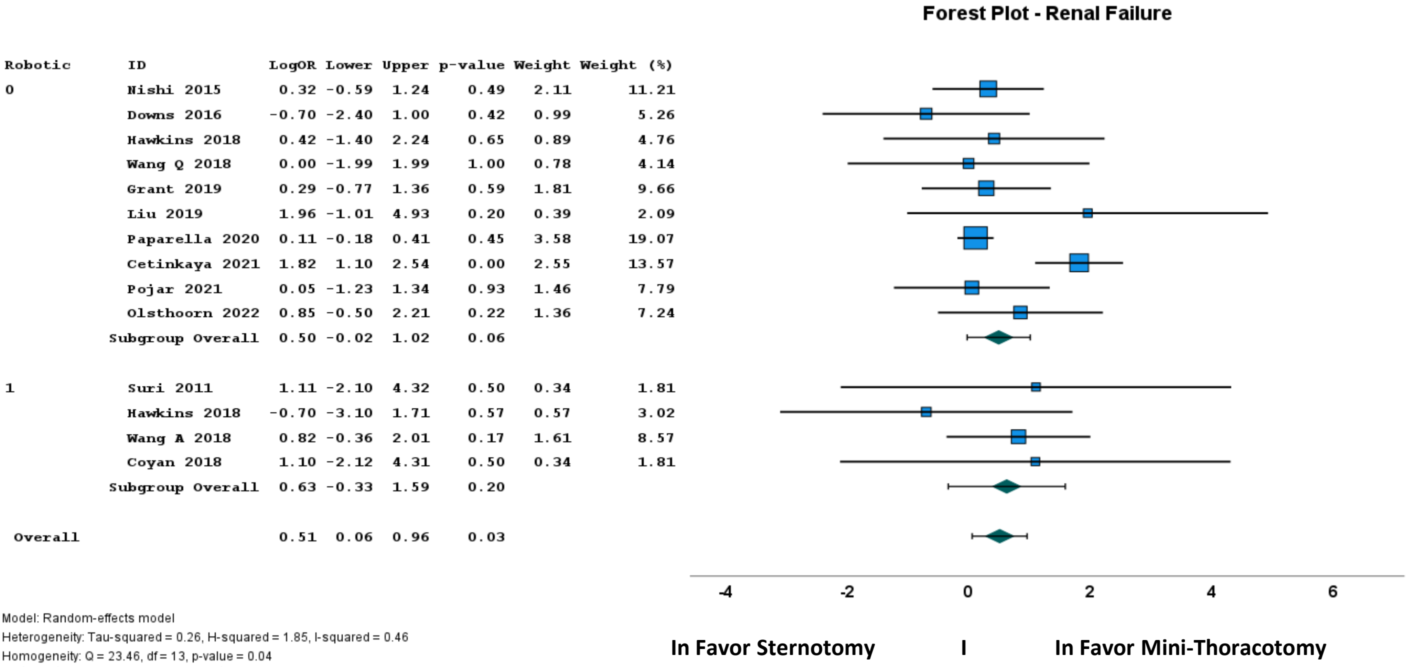

Model: Random-effects model
Heterogeneity: Tau-squared = 0.26, H-squared = 1.85, I-squared = 0.46
Homogeneity: Q = 23.46, df = 13, p-value = 0.04
Test of between-subgroup homogeneity: Q = 0.06, df = 1, p-value = 0.81

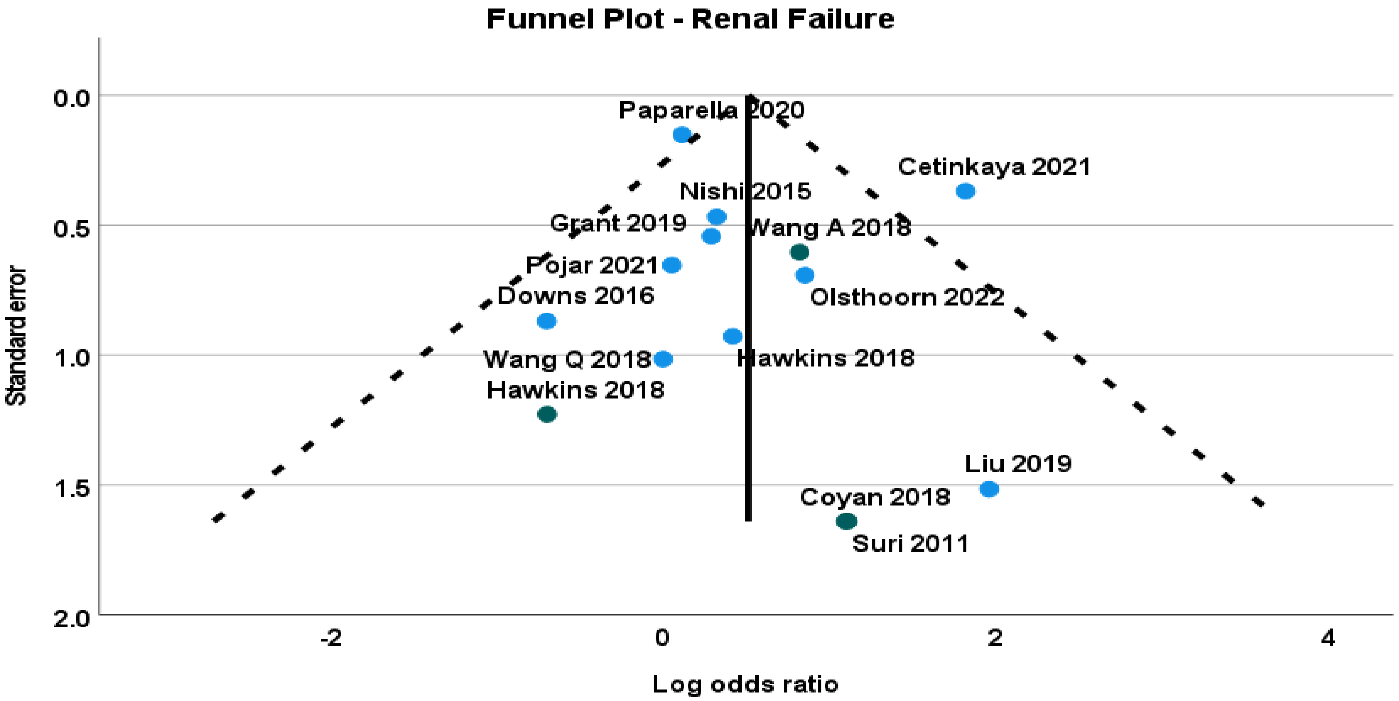

Robotic	ID	LogOR	Lower	Upper	p-value	Weight	Weight (%)
0	Nishi 2015	0.12	-0.15	0.38	0.38	24.85	12.17
	Downs 2016	0.20	-0.19	0.59	0.32	16.07	7.87
	Hawkins 2018	0.16	-0.62	0.94	0.69	5.50	2.69
	Wang Q 2018	0.32	-0.80	1.44	0.57	2.88	1.41
	Liu 2019	0.35	-0.82	1.51	0.56	2.67	1.31
	Paparella 2020	0.12	-0.09	0.32	0.27	30.34	14.86
	Cetinkaya 2021	0.65	0.30	0.99	0.00	18.82	9.22
	Pojar 2021	-0.01	-0.43	0.40	0.95	14.96	7.33
	Olsthoorn 2022	0.46	0.22	0.69	0.00	27.51	13.47
	Subgroup Overall	0.26	0.09	0.43	0.00		
1	Mihaljevic 2011	0.19	-0.41	0.78	0.54	8.68	4.25
	Hawkins 2018	0.15	-0.21	0.50	0.41	18.24	8.93
	Wang A 2018	0.54	0.27	0.80	0.00	24.95	12.22
	Coyan 2018	0.14	-0.46	0.74	0.65	8.67	4.25
	Subgroup Overall	0.31	0.06	0.57	0.02		
Overall		0.27	0.14	0.41	0.00		

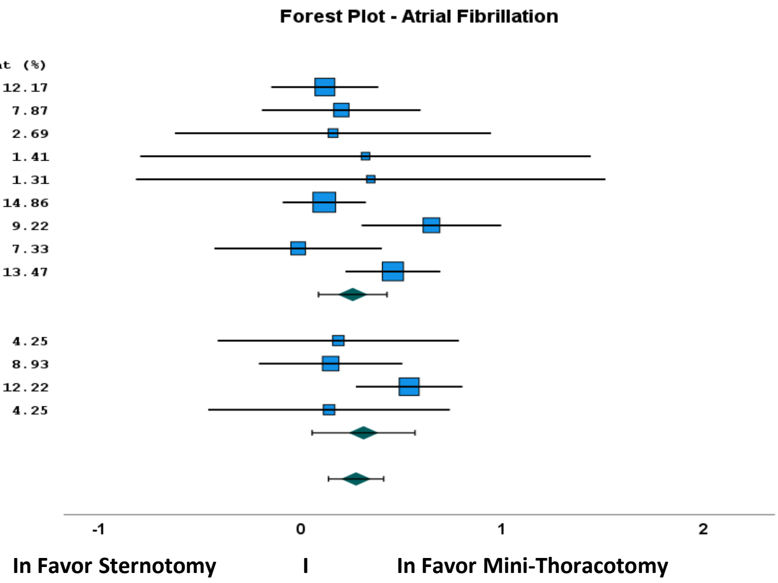

Model: Random-effects model
Heterogeneity: Tau-squared = 0.02, H-squared = 1.65, I-squared = 0.39
Homogeneity: Q = 16.96, df = 12, p-value = 0.15
Test of between-subgroup homogeneity: Q = 0.12, df = 1, p-value = 0.73

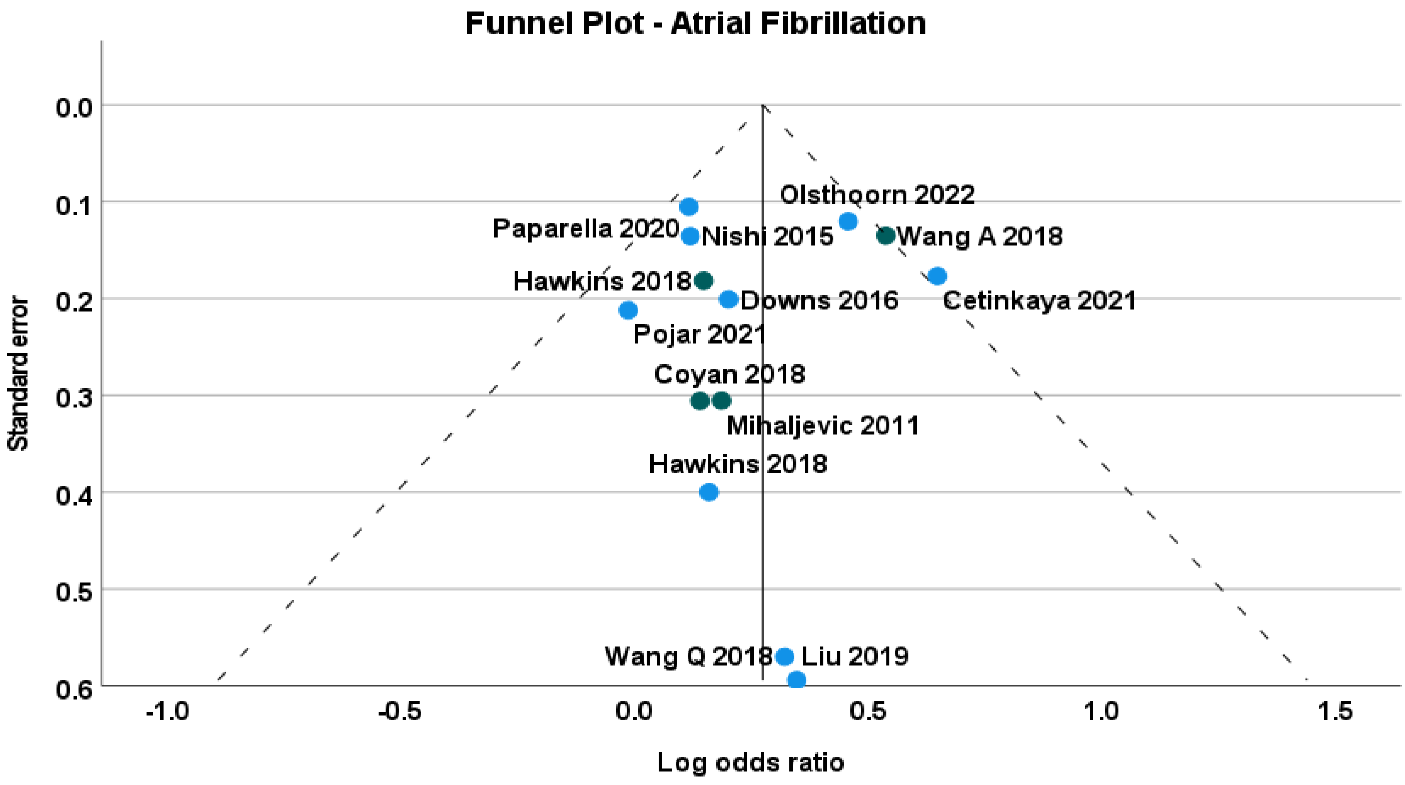

Robotic	ID	LogOR	Lower	Upper	p-value	Weight	Weight (%)
0	Nishi 2015	-0.13	-0.34	0.08	0.24	7.09	11.25
	Downs 2016	1.09	0.69	1.49	0.00	5.85	9.27
	Hawkins 2018	1.12	0.22	2.01	0.01	2.96	4.69
	Wang Q 2018	1.42	0.66	2.18	0.00	3.58	5.67
	Grant 2019	0.43	0.13	0.72	0.00	6.59	10.44
	Liu 2019	0.46	0.03	0.89	0.04	5.60	8.88
	Paparella 2020	0.33	0.17	0.49	0.00	7.35	11.65
	Pojar 2021	0.74	0.30	1.18	0.00	5.56	8.82
	Subgroup Overall	0.60	0.26	0.94	0.00		
1	Mihaljevic 2011	0.78	-0.12	1.67	0.09	2.96	4.69
	Suri 2011	0.63	-0.21	1.46	0.14	3.22	5.10
	Hawkins 2018	0.18	-0.24	0.61	0.39	5.69	9.02
	Wang 1 2018	0.75	0.46	1.03	0.00	6.65	10.54
	Subgroup Overall	0.56	0.21	0.90	0.00		
Overall		0.57	0.33	0.82	0.00		

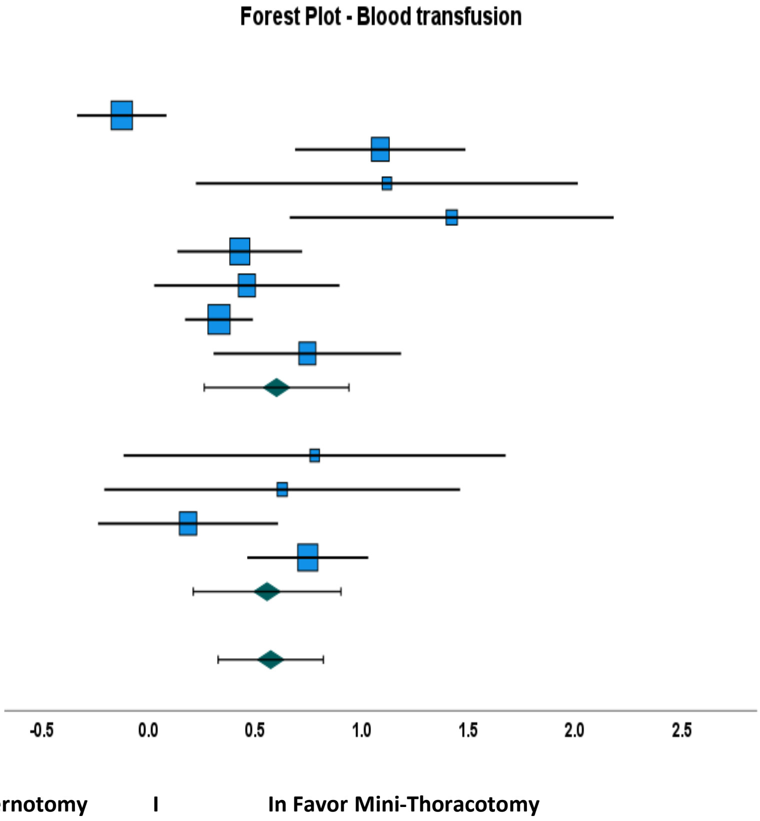

Model: Random-effects model
Heterogeneity: Tau-squared = 0.13, H-squared = 5.18, I-squared = 0.81
Homogeneity: Q = 55.68, df = 11, p-value = 0.00
Test of overall effect size: z = 4.55, p-value = 0.00
Test of between-subgroup homogeneity: Q = 0.03, df = 1, p-value = 0.86

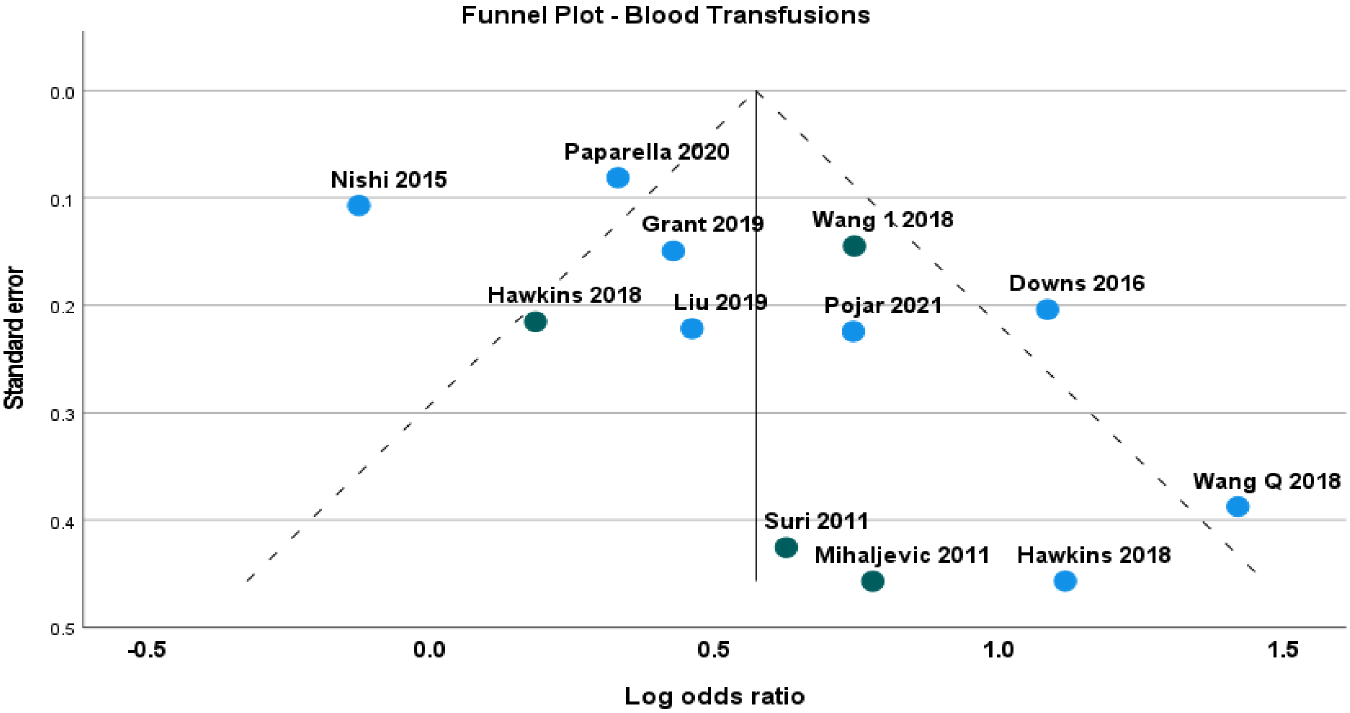

Forest Plot - Prolonged ventilation

Robotic	ID	LogOR	Lower	Upper	p-value	Weight	Weight (%)
0	Nishi 2015	-0.71	-1.47	0.06	0.07	2.80	13.38
	Downs 2016	-0.54	-1.20	0.12	0.11	3.16	15.07
	Hawkins 2018	0.43	-0.87	1.74	0.52	1.54	7.35
	Wang Q 2018	0.54	-0.93	2.02	0.47	1.30	6.21
	Pojar 2021	0.93	0.16	1.71	0.02	2.77	13.25
	Olsthoorn 2022	0.29	-0.77	1.35	0.59	2.01	9.58
	Subgroup Overall	0.08	-0.52	0.68	0.79		
1	Mihaljevic 2011	0.42	-1.39	2.22	0.65	0.95	4.52
	Suri 2011	0.00	-2.79	2.79	1.00	0.45	2.15
	Hawkins 2018	0.34	-0.39	1.07	0.36	2.91	13.91
	Wang A 2018	0.64	-0.05	1.32	0.07	3.06	14.61
	Subgroup Overall	0.48	0.00	0.95	0.05		
Overall		0.20	-0.23	0.63	0.36		

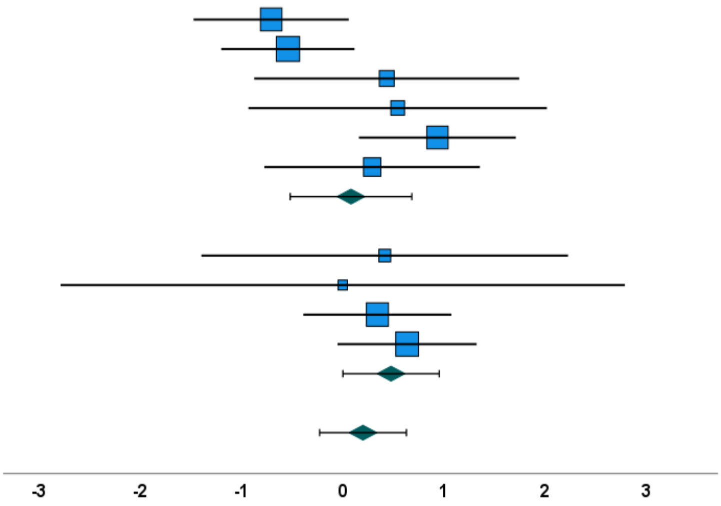

In Favor Sternotomy | In Favor Mini-Thoracotomy

Model: Random-effects model
Heterogeneity: Tau-squared = 0.20, H-squared = 1.90, I-squared = 0.47
Homogeneity: Q = 15.78, df = 9, p-value = 0.07
Test of between-subgroup homogeneity: Q = 1.03, df = 1, p-value = 0.31

Forest Plot - Wound infection

Robotic	ID	LogOR	Lower	Upper	p-value	Weight	Weight (%)
0	Grossi 2014	0.31	-0.32	0.93	0.34	4.73	33.51
	Nishi 2015	1.61	-1.43	4.65	0.30	0.40	2.82
	Downs 2016	0.00	-3.92	3.92	1.00	0.24	1.72
	Hawkins 2018	0.00	-3.93	3.93	1.00	0.24	1.71
	Liu 2019	1.74	0.49	2.99	0.01	1.94	13.77
	Paparella 2020	0.34	-0.33	1.00	0.32	4.46	31.62
	Cetinkaya 2021	0.00	-3.92	3.92	1.00	0.24	1.72
	Pojar 2021	1.76	-0.33	3.84	0.10	0.80	5.69
	Subgroup Overall	0.66	0.09	1.23	0.02		
1	Wang A 2018	1.10	-1.16	3.37	0.34	0.69	4.90
	Coyan 2018	1.11	-2.10	4.32	0.50	0.36	2.53
	Subgroup Overall	1.10	-0.75	2.96	0.24		
Overall		0.68	0.15	1.20	0.01		

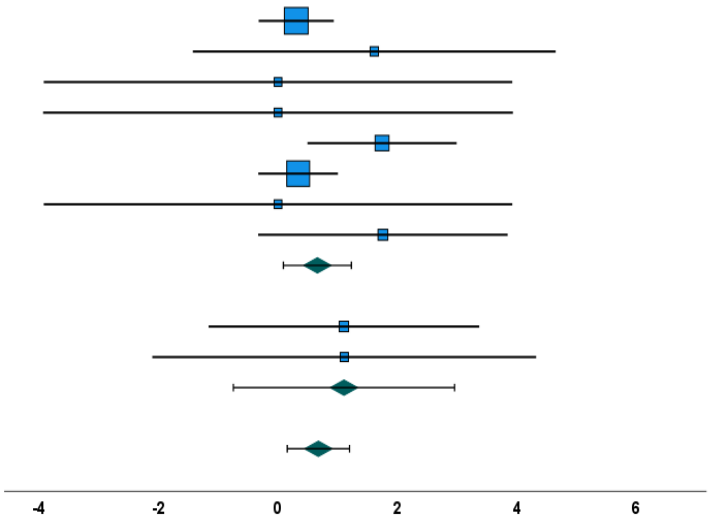

Model: Random-effects model
Heterogeneity: Tau-squared = 0.11, H-squared = 1.20, I-squared = 0.17
Homogeneity: Q = 6.78, df = 9, p-value = 0.66
Test of overall effect size: z = 2.54, p-value = 0.01
Test of between-subgroup homogeneity: Q = 0.20, df = 1, p-value = 0.65

In Favor Sternotomy | In Favor Mini-Thoracotomy

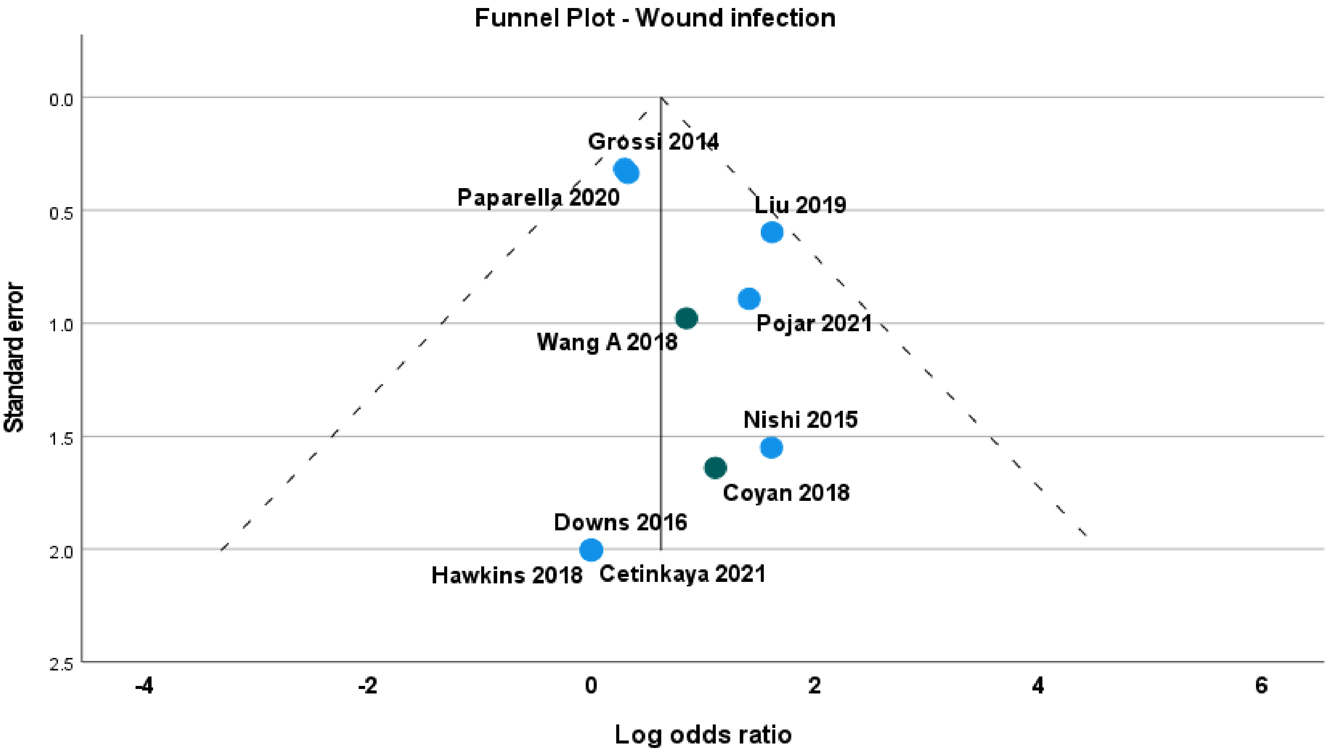

Robotic	ID	LogOR	Lower	Upper	p-value	Weight	Weight (%)
0	Nasso 2014	-0.71	-3.13	1.72	0.57	0.66	2.95
	Wang Q 2018	0.76	-0.67	2.19	0.30	1.88	8.45
	Grant 2019	-0.49	-1.28	0.31	0.23	6.03	27.15
	Liu 2019	0.73	-1.68	3.14	0.55	0.66	2.98
	Pojar 2021	0.08	-0.98	1.13	0.89	3.46	15.57
	Olsthoorn 2022	-0.47	-1.15	0.22	0.18	8.13	36.58
	Subgroup Overall	-0.24	-0.67	0.19	0.27		
1	Wang A 2018	-0.82	-2.48	0.83	0.33	1.41	6.33
	Subgroup Overall	-0.82	-2.48	0.83	0.33		
Overall		-0.28	-0.69	0.14	0.19		

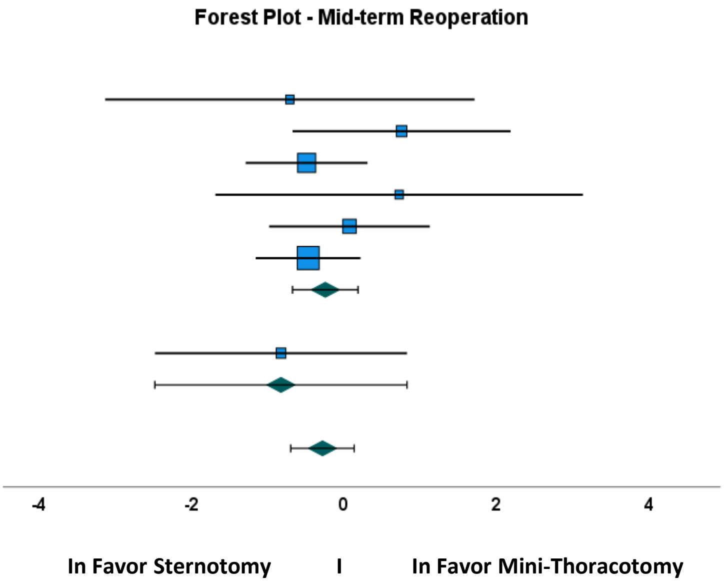

Model: Random-effects model
Heterogeneity: Tau-squared = 0.00, H-squared = 1.00, I-squared = 0.00
Homogeneity: Q = 4.20, df = 6, p-value = 0.65
Test of between-subgroup homogeneity: Q = 0.45, df = 1, p-value = 0.50

Robotic	ID	LogOR	Lower	Upper	p-value	Weight	Weight (%)
0	Nasso 2014	-0.01	-2.80	2.78	0.99	0.48	1.28
	Wang Q 2018	0.00	-3.93	3.93	1.00	0.25	0.65
	Grant 2019	0.73	0.27	1.20	0.00	9.50	25.28
	Liu 2019	0.26	-1.07	1.59	0.70	1.96	5.22
	Cetinkaya 2021	0.64	0.13	1.15	0.01	8.60	22.86
	Pojar 2021	0.45	-0.37	1.27	0.29	4.47	11.89
	Olsthoorn 2022	-0.08	-0.60	0.45	0.78	8.32	22.12
	Subgroup Overall	0.42	0.07	0.78	0.02		
1	Wang A 2018	0.25	-0.63	1.13	0.58	4.02	10.69
	Subgroup Overall	0.25	-0.63	1.13	0.58		
Overall		0.41	0.09	0.73	0.01		

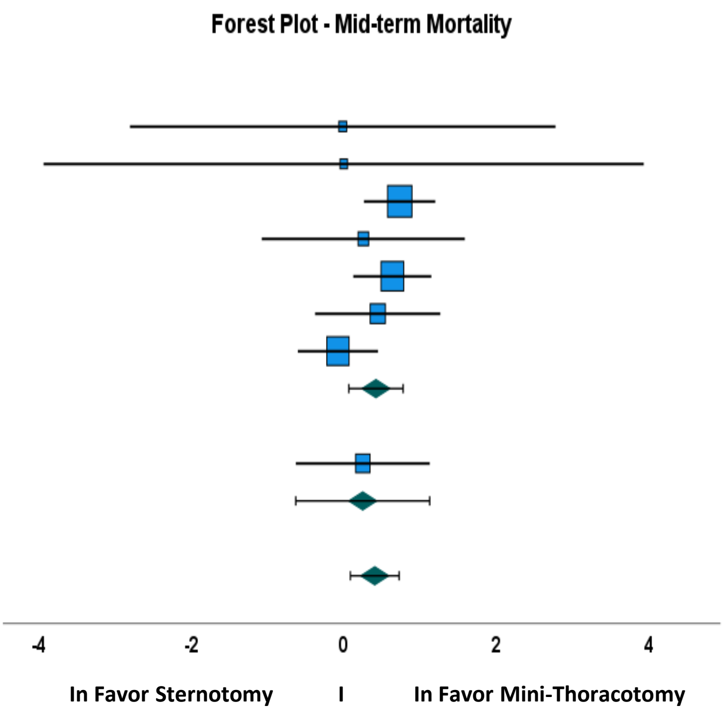

Model: Random-effects model

Heterogeneity: Tau-squared = 0.05, H-squared = 1.32, I-squared = 0.24

Homogeneity: Q = 6.19, df = 7, p-value = 0.52

Test of between-subgroup homogeneity: Q = 0.13, df = 1, p-value = 0.72

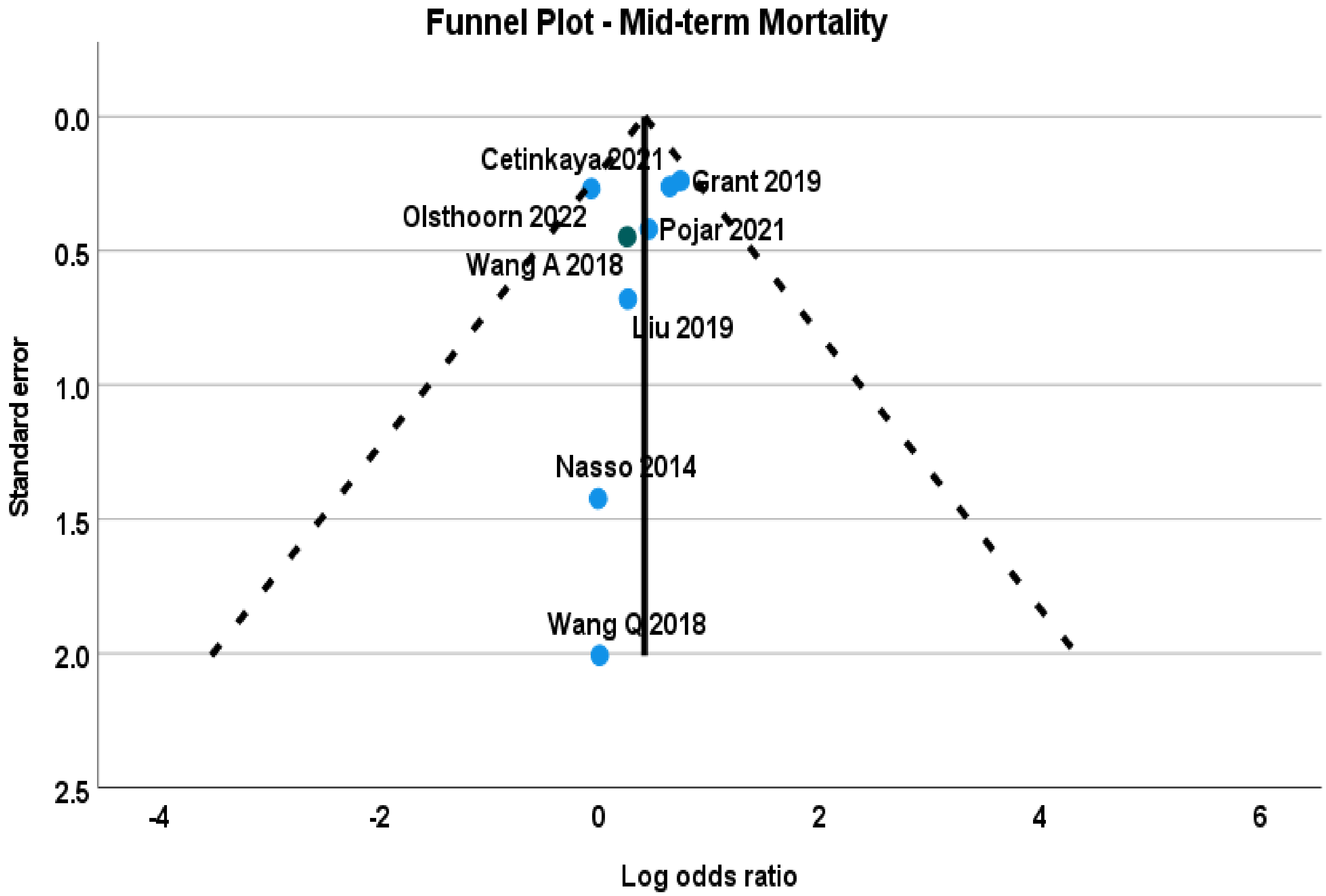

Robotic	ID	Mean difference	Lower	Upper	p-value	Weight	Weight (%)
0	Nasso 2014	22.00	15.63	28.37	0.00	0.01	6.34
	Nishi 2015	30.00	25.65	34.35	0.00	0.01	6.61
	Downs 2016	24.50	19.98	29.02	0.00	0.01	6.59
	Hawkins 2018	21.40	12.97	29.83	0.00	0.01	5.99
	Wang Q 2018	12.90	6.50	19.30	0.00	0.01	6.33
	Grant 2019	28.50	25.44	31.56	0.00	0.01	6.74
	Liu 2019	37.47	33.19	41.75	0.00	0.01	6.62
	Paparella 2020	5.00	2.80	7.20	0.00	0.01	6.80
	Cetinkaya 2021	7.90	4.74	11.06	0.00	0.01	6.73
	Pojar 2021	12.90	7.42	18.38	0.00	0.01	6.47
	Subgroup Overall	20.23	13.62	26.85	0.00		
1	Mihaljevic 2011	28.00	21.35	34.65	0.00	0.01	6.30
	Suri 2011	45.46	38.53	52.39	0.00	0.01	6.25
	Iyigun 2017	10.55	-11.13	32.23	0.34	0.00	3.48
	Hawkins 2018	18.60	14.55	22.65	0.00	0.01	6.65
	Wang A 2018	6.00	1.54	10.46	0.01	0.01	6.60
	Coyan 2018	16.00	4.96	27.04	0.00	0.01	5.49
	Subgroup Overall	21.37	9.56	33.17	0.00		
Overall		20.68	14.92	26.45	0.00		

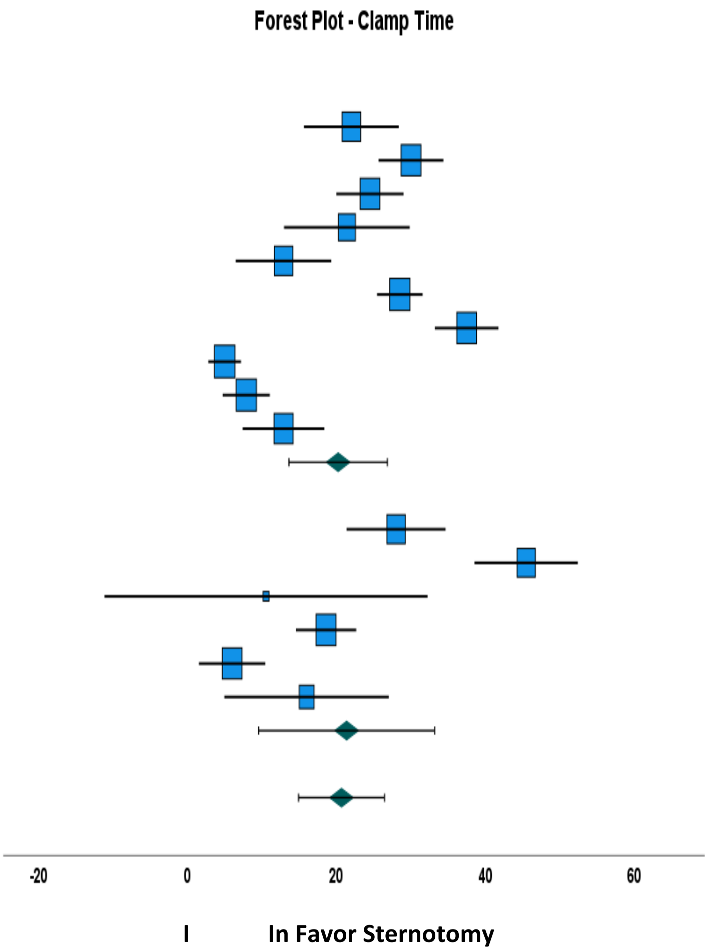

Model: Random-effects model

Heterogeneity: Tau-squared = 125.87, H-squared = 24.60, I-squared = 0.96

Homogeneity: Q = 439.18, df = 15, p-value = 0.00

Test of overall effect size: z = 7.03, p-value = 0.00

Test of between-subgroup homogeneity: Q = 0.03, df = 1, p-value = 0.87

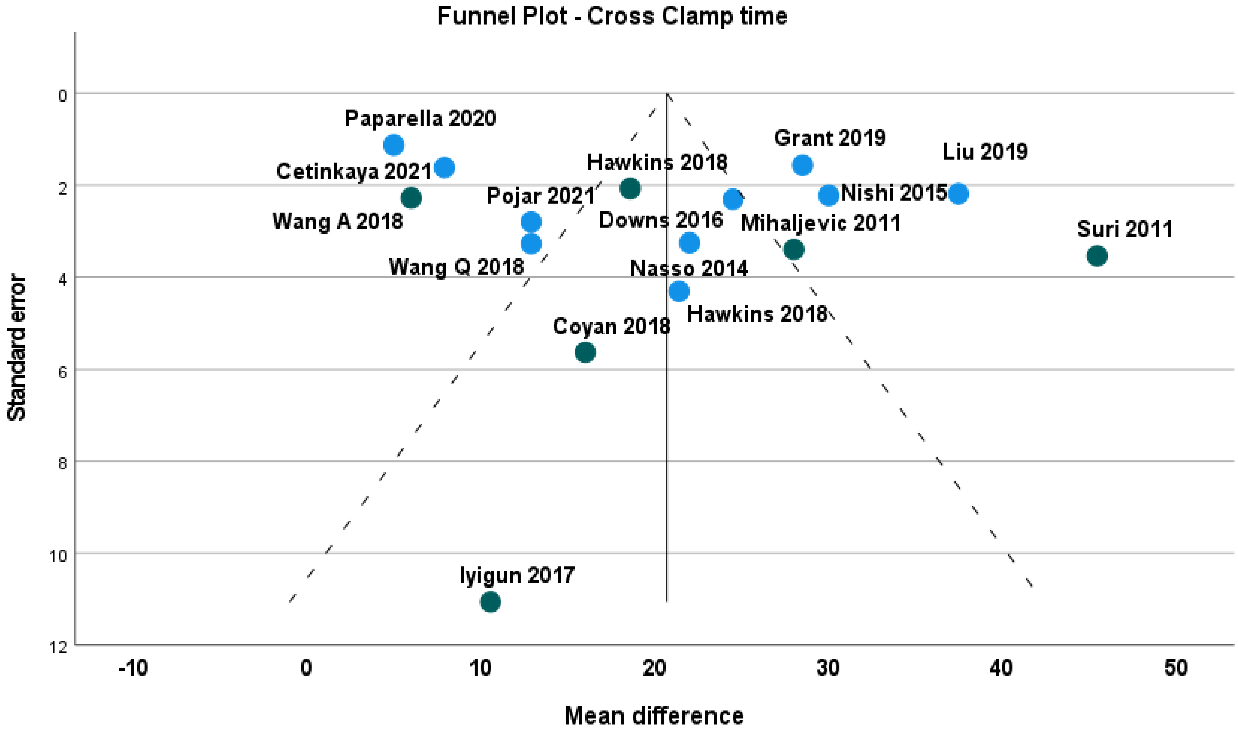

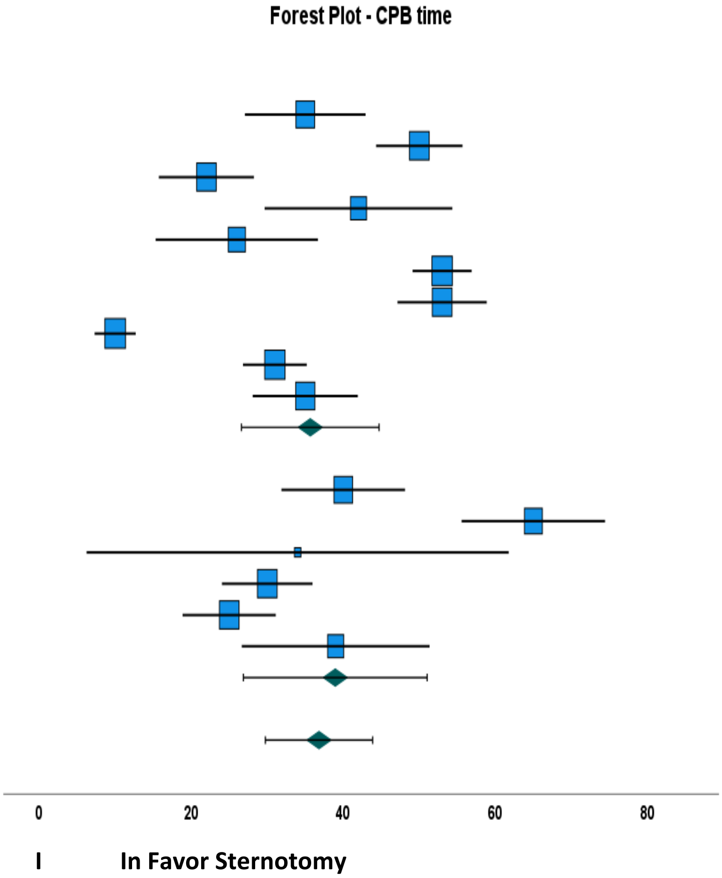

Robotic	ID	Mean difference	Lower	Upper	p-value	Weight	Weight (%)
0	Nasso 2014	35.00	27.06	42.94	0.00	0.00	6.40
	Nishi 2015	50.00	44.32	55.68	0.00	0.01	6.66
	Downs 2016	22.00	15.75	28.25	0.00	0.01	6.60
	Hawkins 2018	42.00	29.65	54.35	0.00	0.00	5.74
	Wang Q 2018	26.00	15.33	36.67	0.00	0.00	6.01
	Grant 2019	53.00	49.11	56.89	0.00	0.01	6.82
	Liu 2019	53.00	47.13	58.87	0.00	0.01	6.64
	Paparella 2020	10.00	7.29	12.71	0.00	0.01	6.89
	Cetinkaya 2021	31.00	26.80	35.20	0.00	0.01	6.80
	Pojar 2021	35.00	28.09	41.91	0.00	0.01	6.53
	Subgroup Overall	35.66	26.62	44.71	0.00		
1	Mihaljevic 2011	40.00	31.87	48.13	0.00	0.00	6.37
	Suri 2011	65.00	55.56	74.44	0.00	0.00	6.19
	Iyigun 2017	34.00	6.24	61.76	0.02	0.00	3.35
	Hawkins 2018	30.00	24.04	35.96	0.00	0.01	6.63
	Wang A 2018	25.00	18.87	31.13	0.00	0.01	6.62
	Coyan 2018	39.00	26.65	51.35	0.00	0.00	5.74
	Subgroup Overall	38.95	26.87	51.04	0.00		
Overall		36.81	29.75	43.87	0.00		

Model: Random-effects model
Heterogeneity: Tau-squared = 186.19, H-squared = 21.09, I-squared = 0.95
Homogeneity: Q = 524.67, df = 15, p-value = 0.00
Test of overall effect size: z = 10.22, p-value = 0.00
Test of between-subgroup homogeneity: Q = 0.18, df = 1, p-value = 0.67

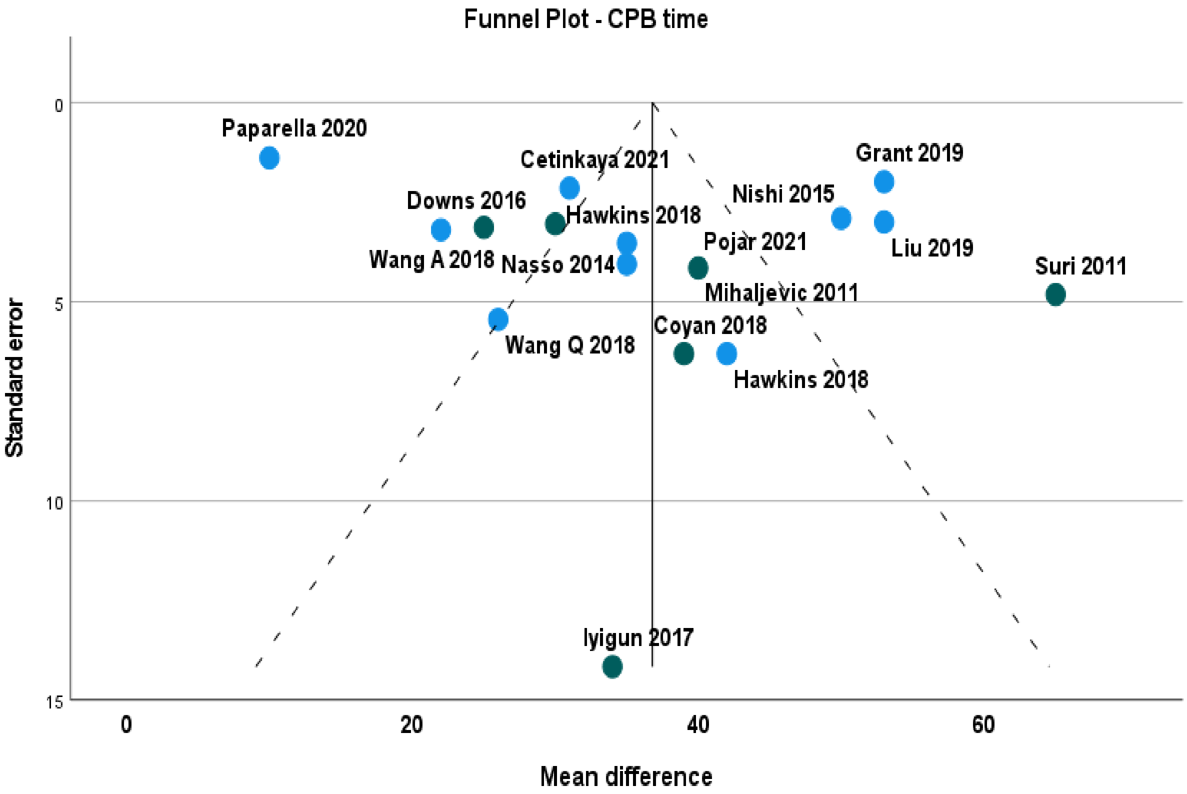

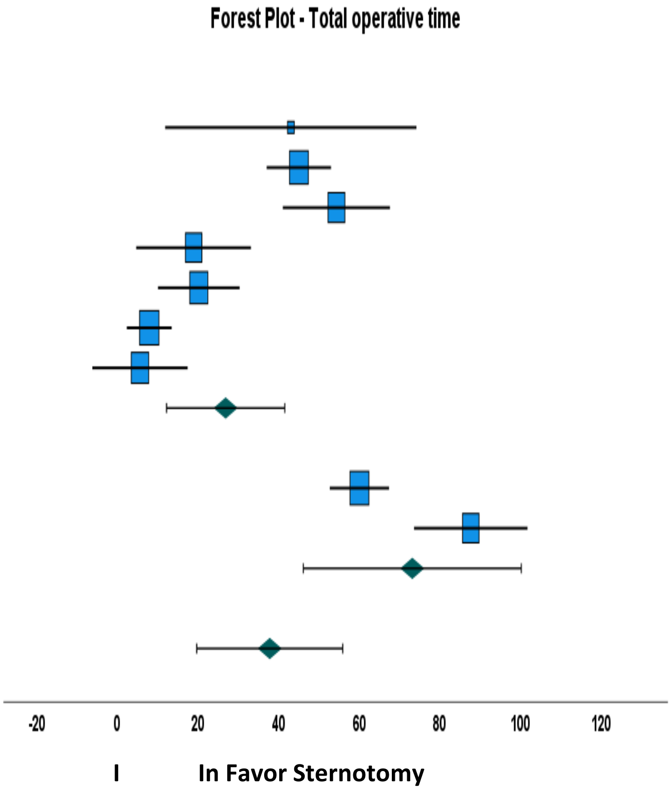

Robotic	ID	Mean difference	Lower	Upper	p-value	Weight	Weight (%)
0	Nasso 2014	43.00	11.86	74.14	0.01	0.00	8.80
	Nishi 2015	45.00	37.03	52.97	0.00	0.00	11.63
	Hawkins 2018	54.30	41.01	67.59	0.00	0.00	11.18
	Wang 2018	18.90	4.68	33.12	0.01	0.00	11.08
	Liu 2019	20.18	10.06	30.30	0.00	0.00	11.47
	Cetinkaya 2021	7.90	2.34	13.46	0.01	0.00	11.76
	Pojar 2021	5.60	-6.22	17.42	0.35	0.00	11.32
	Subgroup Overall	26.85	12.19	41.50	0.00		
1	Mihaljevic 2011	60.00	52.65	67.35	0.00	0.00	11.67
	Hawkins 2018	87.60	73.53	101.67	0.00	0.00	11.10
	Subgroup Overall	73.12	46.11	100.14	0.00		
Overall		37.78	19.68	55.87	0.00		

Model: Random-effects model
Heterogeneity: Tau-squared = 716.35, H-squared = 29.06, I-squared = 0.97
Homogeneity: Q = 237.11, df = 8, p-value = 0.00
Test of overall effect size: z = 4.09, p-value = 0.00
Test of between-subgroup homogeneity: Q = 8.71, df = 1, p-value = 0.00

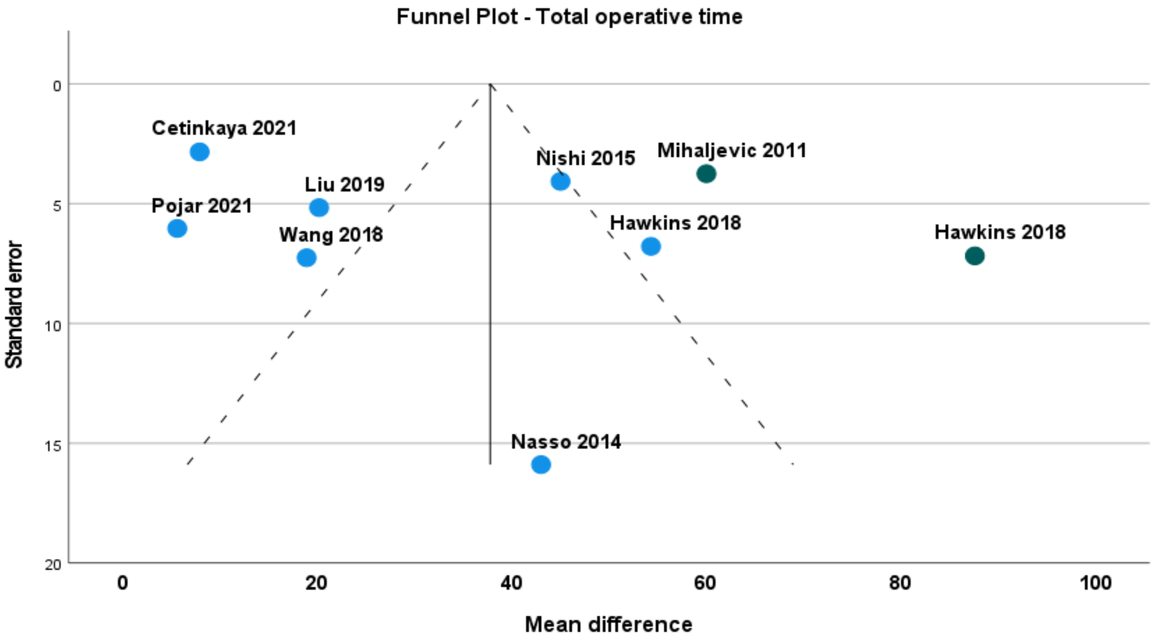

Forest Plot - Ventilation time

Robotic	ID	Mean difference	Lower	Upper	p-value	Weight	Weight (%)
0	Nasso 2014	-24.00	-27.53	-20.47	0.00	0.01	12.15
	Downs 2016	-0.56	-0.90	-0.22	0.00	0.01	12.67
	Hawkins 2018	-3.60	-4.95	-2.25	0.00	0.01	12.60
	Wang 2018	0.60	-0.03	1.23	0.06	0.01	12.66
	Liu 2019	-6.08	-9.14	-3.02	0.00	0.01	12.28
	Cetinkaya 2021	-2.00	-2.61	-1.39	0.00	0.01	12.66
	Pojar 2021	-7.90	-9.99	-5.81	0.00	0.01	12.49
	Subgroup Overall	-6.10	-12.19	-0.01	0.05		
1	Suri 2011	5.94	3.77	8.11	0.00	0.01	12.48
	Subgroup Overall	5.94	3.77	8.11	0.00		
Overall		-4.61	-10.65	1.43	0.13		

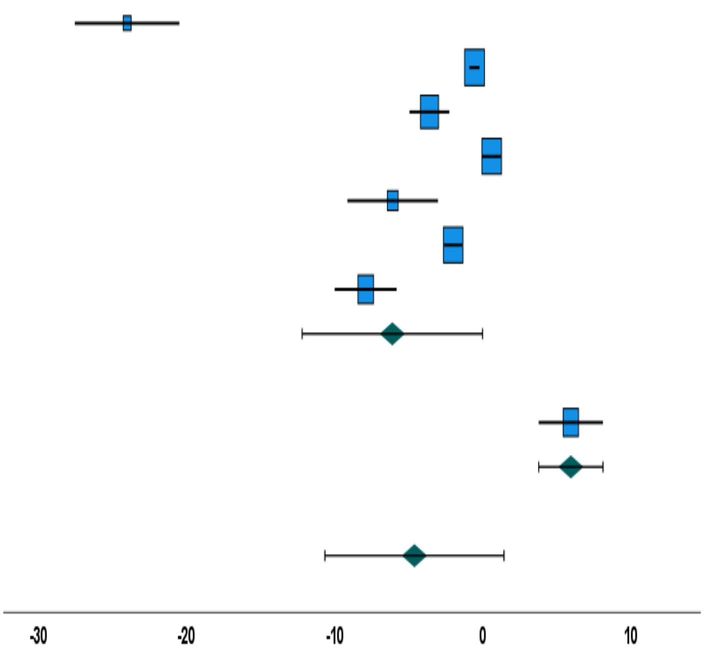

In Favor Mini-Thoracotomy I

Model: Random-effects model

Heterogeneity: Tau-squared = 74.92, H-squared = 374.59, I-squared = 1.00

Homogeneity: Q = 310.23, df = 7, p-value = 0.00

Test of overall effect size: z = -1.50, p-value = 0.13

Test of between-subgroup homogeneity: Q = 13.33, df = 1, p-value = 0.00

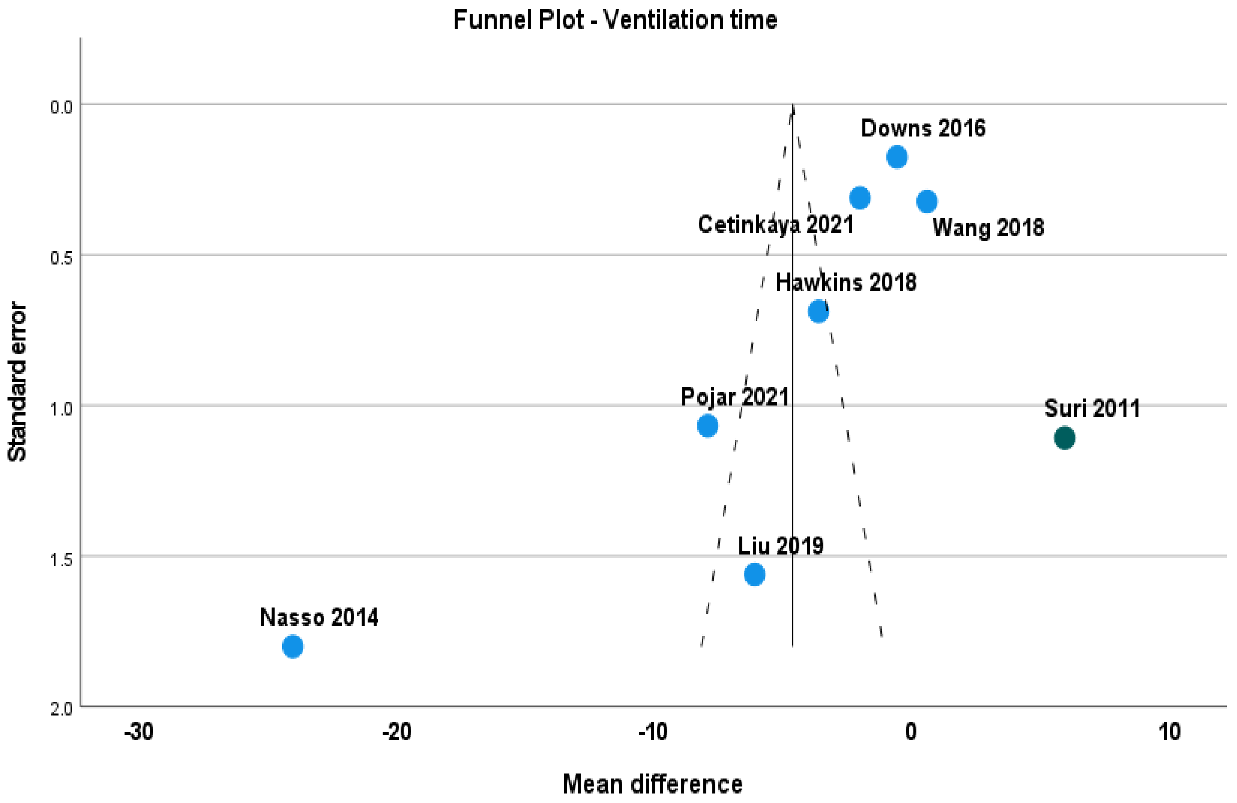

Robotic	ID	Mean difference	Lower	Upper	p-value	Weight	Weight (%)
0	Nasso 2014	-1.10	-1.43	-0.77	0.00	1.80	7.53
	Nishi 2015	-0.20	-0.55	0.15	0.26	1.78	7.48
	Downs 2016	-0.57	-0.76	-0.38	0.00	1.86	7.79
	Hawkins 2018	-0.38	-1.02	0.26	0.25	1.57	6.58
	Wang Q 2018	-0.58	-0.68	-0.48	0.00	1.88	7.89
	Liu 2019	-0.70	-1.35	-0.05	0.03	1.57	6.57
	Paparella 2020	-0.03	-0.08	0.02	0.27	1.89	7.91
	Cetinkaya 2021	-0.21	-0.36	-0.06	0.01	1.87	7.84
	Pojar 2021	-0.50	-1.36	0.36	0.25	1.39	5.83
	Subgroup Overall	-0.45	-0.69	-0.22	0.00		
1	Suri 2011	0.22	-0.70	1.14	0.64	1.34	5.61
	Iyigun 2017	-0.16	-0.46	0.14	0.30	1.81	7.58
	Hawkins 2018	-0.45	-0.63	-0.27	0.00	1.86	7.80
	Wang A 2018	-0.75	-1.02	-0.48	0.00	1.82	7.64
	Coyan 2018	-3.50	-4.32	-2.68	0.00	1.42	5.95
	Subgroup Overall	-0.91	-2.16	0.34	0.15		
Overall		-0.61	-1.01	-0.21	0.00		

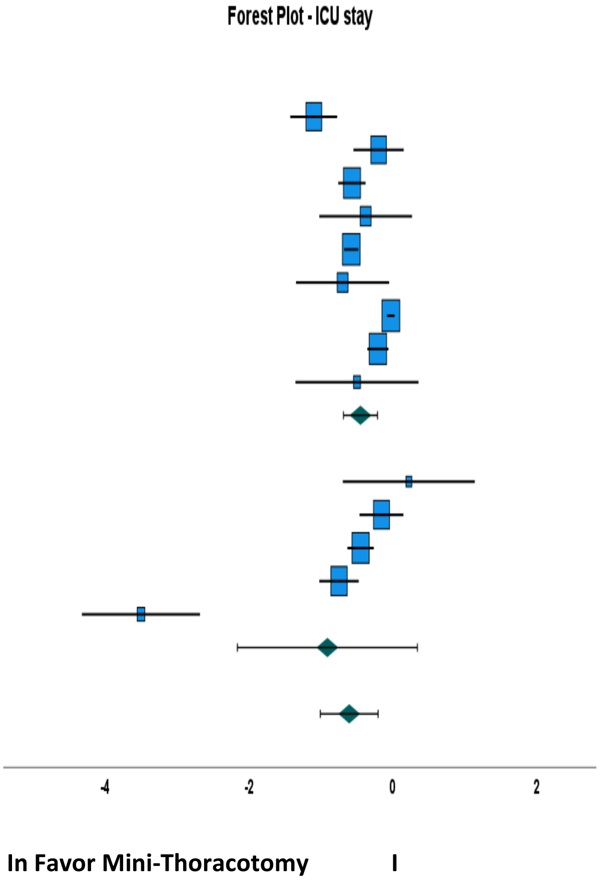

Model: Random-effects model
Heterogeneity: Tau-squared = 0.53, H-squared = 60.53, I-squared = 0.98
Homogeneity: Q = 226.51, df = 13, p-value = 0.00
Test of overall effect size: z = -2.97, p-value = 0.00
Test of between-subgroup homogeneity: Q = 0.50, df = 1, p-value = 0.48

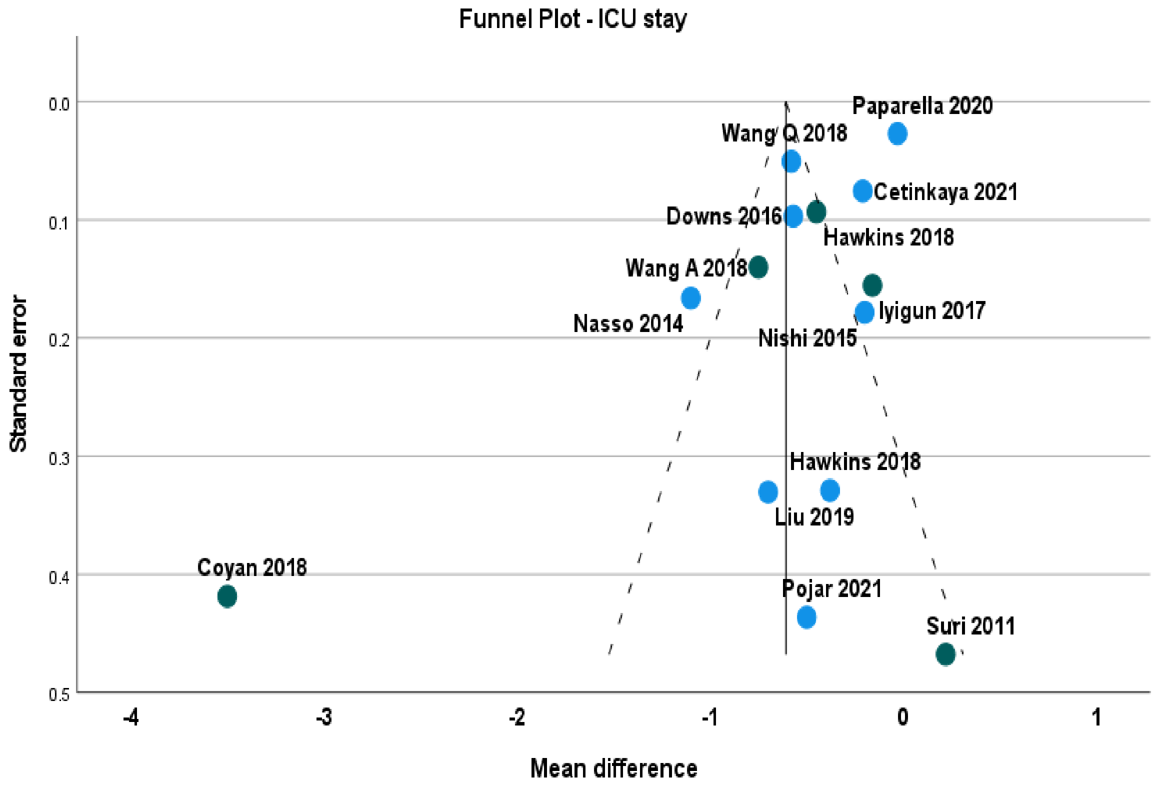

Forest Plot - Hospital stay

Robotic	ID	Mean difference	Lower	Upper	p-value	Weight	Weight (%)
0	Nasso 2014	-3.10	-4.57	-1.63	0.00	0.30	5.36
	Nishi 2015	-3.00	-4.02	-1.98	0.00	0.33	5.88
	Downs 2016	-1.33	-1.71	-0.95	0.00	0.35	6.36
	Hawkins 2018	0.00	-0.84	0.84	1.00	0.34	6.05
	Wang Q 2018	-3.70	-4.96	-2.44	0.00	0.31	5.61
	Grant 2019	-1.60	-1.92	-1.28	0.00	0.35	6.38
	Liu 2019	-5.80	-6.82	-4.78	0.00	0.33	5.88
	Paparella 2020	-1.00	-1.29	-0.71	0.00	0.35	6.40
	Cetinkaya 2021	-1.70	-2.36	-1.04	0.00	0.34	6.19
	Pojar 2021	0.10	-1.75	1.95	0.92	0.27	4.89
	Olsthoorn 2022	0.30	0.03	0.57	0.03	0.36	6.40
	Subgroup Overall	-1.87	-2.96	-0.79	0.00		
1	Mihaljevic 2011	-1.00	-2.03	0.03	0.06	0.33	5.87
	Suri 2011	-0.88	-2.21	0.45	0.19	0.31	5.54
	Iyigun 2017	-2.00	-3.25	-0.75	0.00	0.31	5.63
	Hawkins 2018	-0.70	-1.00	-0.40	0.00	0.35	6.39
	Wang A 2018	-1.70	-2.09	-1.31	0.00	0.35	6.36
	Coyan 2018	-6.00	-7.91	-4.09	0.00	0.27	4.81
	Subgroup Overall	-1.91	-3.31	-0.51	0.01		
Overall		-1.88	-2.72	-1.05	0.00		

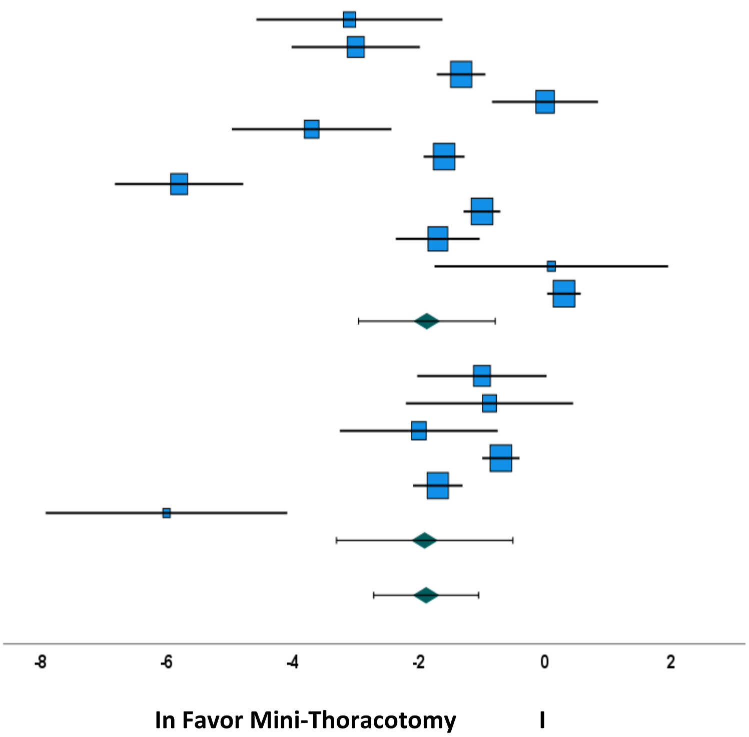

Model: Random-effects model
Heterogeneity: Tau-squared = 2.80, H-squared = 41.34, I-squared = 0.98
Homogeneity: Q = 289.00, df = 16, p-value = 0.00
Test of between-subgroup homogeneity: Q = 0.00, df = 1, p-value = 0.97

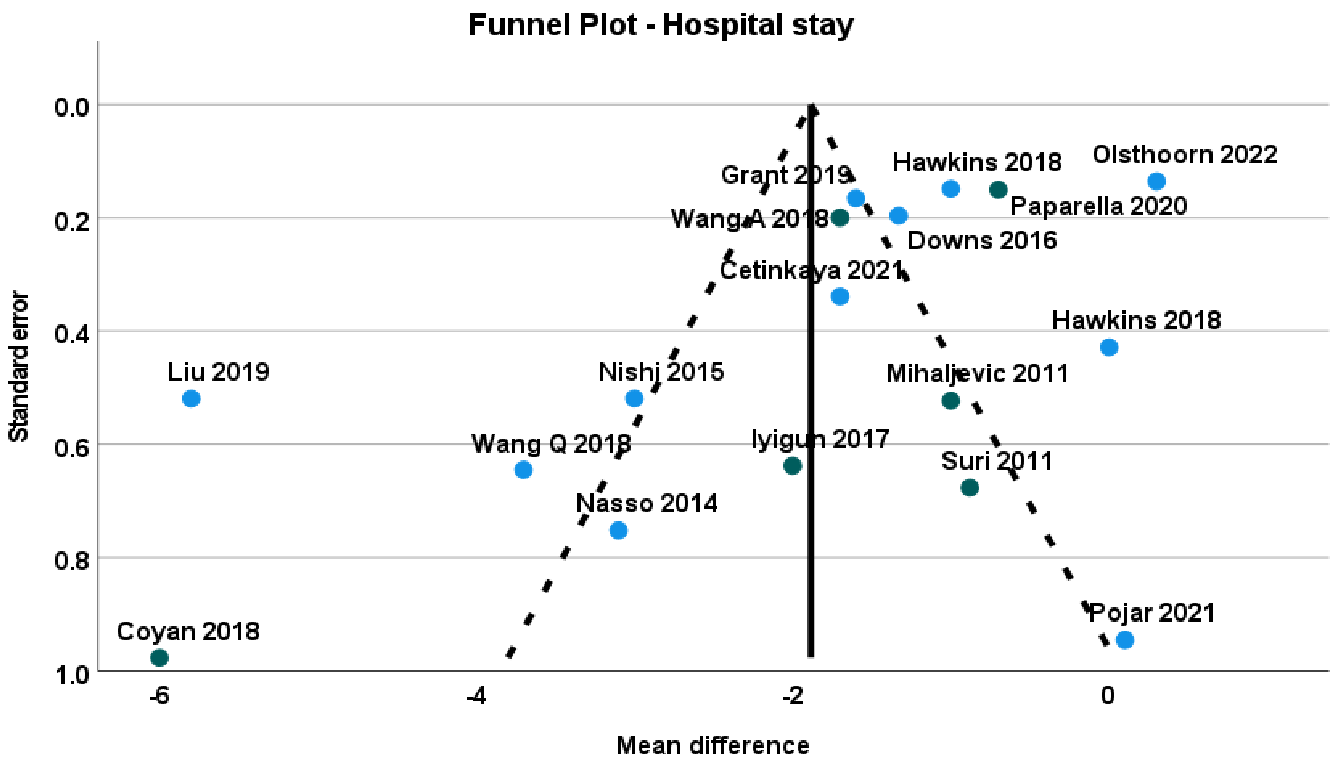

Robotic	ID	Mean difference	Lower	Upper	p-value	Weight	Weight (%)
0	Grossi 2014	-10527.00	-14709.69	-6344.31	0.00	0.00	25.05
	Downs 2016	-3276.00	-4747.12	-1804.88	0.00	0.00	32.02
	Hawkins 2018	-5267.00	-25081.69	14547.69	0.60	0.00	3.96
	Pojar 2021	-904.00	-16518.82	14710.82	0.91	0.00	5.94
	Subgroup Overall	-5890.34	-11263.21	-517.47	0.03		
1	Coyan 2018	-1750.00	-2433.91	-1066.09	0.00	0.00	33.04
	Subgroup Overall	-1750.00	-2433.91	-1066.09	0.00		
Overall		-4525.81	-8725.02	-326.59	0.03		

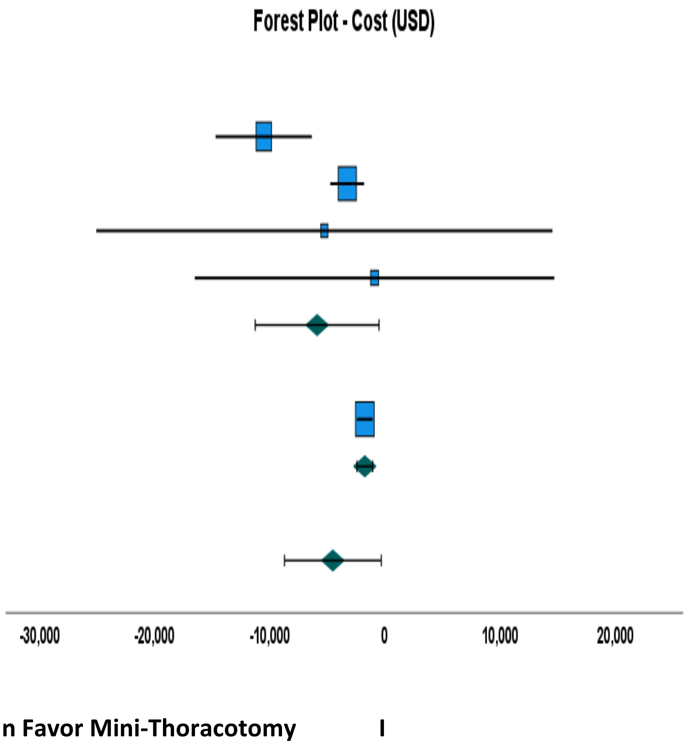

Model: Random-effects model
Heterogeneity: Tau-squared = 13773191.05, H-squared = 12.46, I-squared = 0.92
Homogeneity: Q = 19.06, df = 4, p-value = 0.00
Test of overall effect size: z = -2.11, p-value = 0.03
Test of between-subgroup homogeneity: Q = 2.24, df = 1, p-value = 0.13

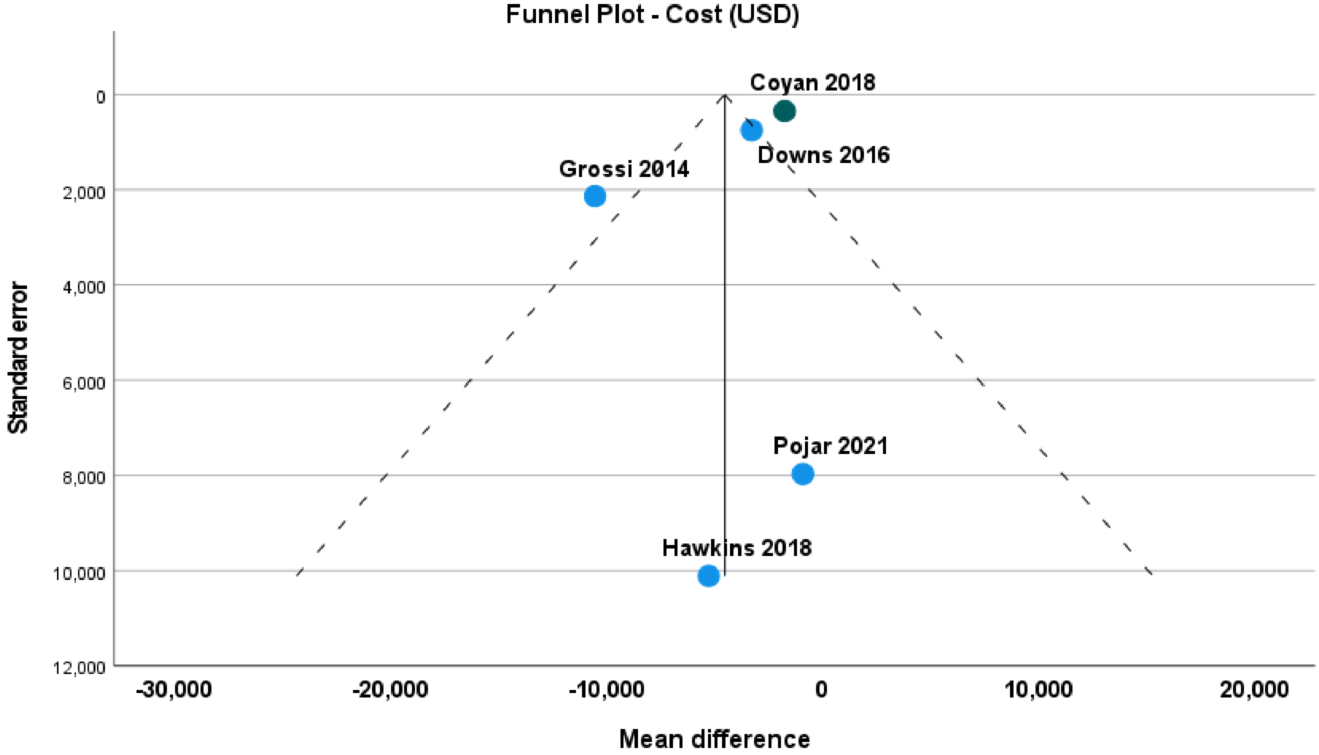

Supplement: Supplementary file 2 — Additional file 2. Forest plots of the criteria analyzed with respective funnel plots if significant Odds ratio. [file 13019_2023_2229_MOESM2_ESM.pdf]
